# Complete genome sequence and metabolic potential of the quinaldine-degrading bacterium *Arthrobacter* sp. Rue61a

**DOI:** 10.1186/1471-2164-13-534

**Published:** 2012-10-06

**Authors:** Heiko Niewerth, Jörg Schuldes, Katja Parschat, Patrick Kiefer, Julia A Vorholt, Rolf Daniel, Susanne Fetzner

**Affiliations:** 1 Institute of Molecular Microbiology and Biotechnology, University of Münster, Corrensstrasse 3, 48149, Münster, Germany; 2 Department of Genomic and Applied Microbiology & Göttingen Genomics Laboratory, Institute of Microbiology and Genetics, Georg-August University Göttingen, 37077, Göttingen, Germany; 3 Institute of Microbiology, ETH Zurich, Zurich, Switzerland; 4 Present address: Jennewein Biotechnologie GmbH, 53619, Rheinbreitbach, Germany

**Keywords:** *Arthrobacter* sp., Soil bacterium, Saprophyte, Biodegradation, 2-Methylquinoline, Heavy metal resistance

## Abstract

**Background:**

Bacteria of the genus *Arthrobacter* are ubiquitous in soil environments and can be considered as true survivalists. *Arthrobacter* sp. strain Rue61a is an isolate from sewage sludge able to utilize quinaldine (2-methylquinoline) as sole carbon and energy source. The genome provides insight into the molecular basis of the versatility and robustness of this environmental *Arthrobacter* strain.

**Results:**

The genome of *Arthrobacter* sp. Rue61a consists of a single circular chromosome of 4,736,495 bp with an average G + C content of 62.32%, the circular 231,551-bp plasmid pARUE232, and the linear 112,992-bp plasmid pARUE113 that was already published. Plasmid pARUE232 is proposed to contribute to the resistance of *Arthrobacter* sp. Rue61a to arsenate and Pb^2+^, whereas the linear plasmid confers the ability to convert quinaldine to anthranilate. Remarkably, degradation of anthranilate exclusively proceeds via a CoA-thioester pathway. Apart from quinaldine utilization, strain Rue61a has a limited set of aromatic degradation pathways, enabling the utilization of 4-hydroxy-substituted aromatic carboxylic acids, which are characteristic products of lignin depolymerization, via *ortho* cleavage of protocatechuate. However, 4-hydroxyphenylacetate degradation likely proceeds via *meta* cleavage of homoprotocatechuate. The genome of strain Rue61a contains numerous genes associated with osmoprotection, and a high number of genes coding for transporters. It encodes a broad spectrum of enzymes for the uptake and utilization of various sugars and organic nitrogen compounds. *A*. *aurescens* TC-1 is the closest sequenced relative of strain Rue61a.

**Conclusions:**

The genome of *Arthrobacter* sp. Rue61a reflects the saprophytic lifestyle and nutritional versatility of the organism and a strong adaptive potential to environmental stress. The circular plasmid pARUE232 and the linear plasmid pARUE113 contribute to heavy metal resistance and to the ability to degrade quinaldine, respectively.

## Background

Strains of *Arthrobacter* species are among the predominant members of culturable aerobic soil bacteria and are thought to play a significant role in the biodegradation of organic matter [1]. They have been detected in the deep subsurface and in extreme environments [2-4] and appear to be abundant in heavy metal-contaminated sites [5-9]. *Arthrobacter* spp. also contribute to the bacterial community in activated sludge of wastewater treatment systems [10-12]; under conditions of unstable organic loading, *Arthrobacter* sp. and other Gram-positives with a rod-coccus cycle were even found to be prevalent [12]. The ubiquity of *Arthrobacter* strains is considered to be due to their nutritional versatility and their pronounced resistance to desiccation, long-term starvation, and environmental stress [1,13,14]. A number of *Arthrobacter* strains harbor plasmids, which contribute to heavy metal resistance or confer catabolic traits [15-17].

The complete genome sequences of five environmental *Arthrobacter* species are available. *A*. *aurescens* TC1, *A*. *chlorophenolicus* A6 and *A*. *phenanthrenivorans* Sphe3 were isolated from soil for their ability to degrade atrazine, 4-chlorophenol, and phenanthrene, respectively [18-21], and the type strain of *A*. *globiformis* (NBRC 12137, ATCC 8010) also is a soil isolate [22]. *Arthrobacter* sp. FB24 was obtained from a microcosm that contained chromate, lead- and hydrocarbon-contaminated soils [15,23]. Genome analyses indicated that soil isolates like strains TC1 and FB24 have a large number of genes encoding stress-related proteins. As expected, the metabolic diversity and niche specialization of the environmental *Arthrobacter* strains is reflected in their genomes. *A*. *aurescens* TC1, for example, appears specialized with respect to its ability to utilize a broad variety of amines and other nitrogenous compounds. Carbohydrate polymers are another metabolic niche of both *A*. *aurescens* TC1 and *Arthrobacter* sp. FB24 [18]. In contrast to these environmental *Arthrobacter* strains, *A*. *arilaitensis* Re117 is an isolate from the surface of cheese, characterized by efficient iron acquisition and salt-tolerance systems and the ability to utilize carbon substrates present in cheese such as lactic acid and fatty acids [24].

*Arthrobacter* sp. strain Rue61a was previously isolated from sludge of the biological wastewater treatment plant of a coal tar refinery in Castrop-Rauxel, Germany, based on its ability to utilize quinaldine (2-methylquinoline) as source of carbon and energy [25,26]. Methylquinolines, quinoline and other *N*-heteroaromatic compounds are constituents of shale oil and coal tar. Since many quinoline derivatives are considered toxic and/or mutagenic, they are of environmental concern. Quinolines are more polar than their homocyclic naphthalene analogs, consequently they are more readily transported to subsoil and groundwater if entering the environment, e.g., from wood-creosoting activities or abandoned coal and oil processing facilities. A number of bacterial isolates, mainly aerobes from soil, with the ability to degrade certain quinoline derivatives have been described in the literature (reviewed in [27]).

In the upper part of the quinaldine degradation pathway of *Arthrobacter* sp. strain Rue61a, quinaldine is oxidized to carbon monoxide, acetate, and anthranilate [28-31]. The genes coding for the enzymes of the “upper pathway” are clustered on a conjugative plasmid, previously termed pAL1, which in contrast to other *Arthrobacter* plasmids described until now has a linear topology [29,32,33]. Anthranilate has been proposed to be metabolized via catechol and subsequent intradiol cleavage, or via a CoA-thioester pathway involving anthranilate CoA-ligase and a putative anthraniloyl-CoA monooxygenase/reductase encoded on the pAL1 plasmid [29]. However, the full catabolic potential of *Arthrobacter* strain Rue61a has not yet been characterized. We therefore analyzed the complete genome of the strain and performed physiological tests as well as an analysis of the CoA-metabolome to complement the genome-based reconstruction of metabolic pathways. The availability of several *Arthrobacter* genomes provides the opportunity for comparative studies towards a better understanding of the molecular basis of the versatility and environmental robustness of this genus.

## Results and discussion

### General genome features and comparative genomics

**Figure 1:**
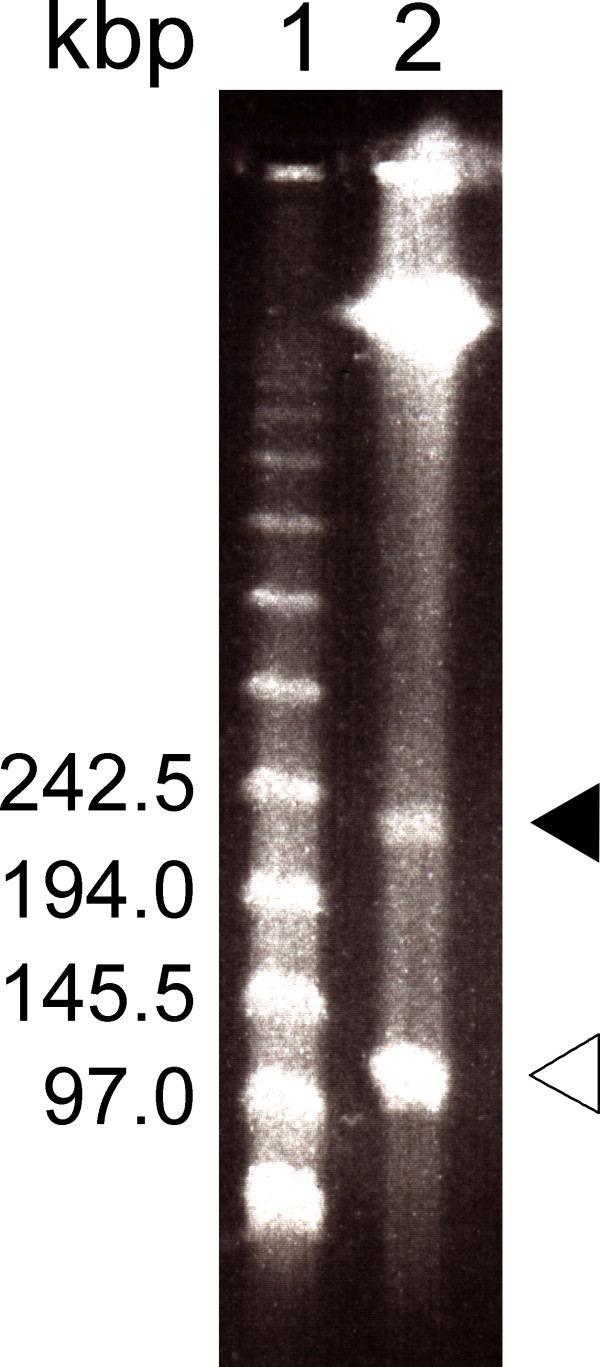
**Separation of total DNA of *Arthrobacter *sp. Rue61a by pulsed-field gel electrophoresis.** Lane 1: Concatemers of bacteriophage λ DNA; lane 2: DNA from *Arthrobacter* sp. Rue61a cells, embedded in agarose plugs and treated with proteinase K after lysis. Filled and open arrowheads indicate the plasmids pARUE232 and pARUE113, respectively.

**Figure 2:**
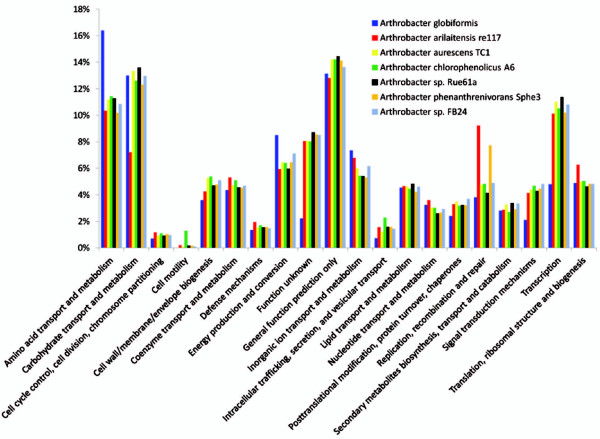
**Percentage of genes assigned to the COG categories in all sequenced *Arthrobacter *species.**

The genome of *Arthrobacter* sp. Rue61a comprises a circular chromosome of 4,736,495 bp, one circular plasmid pARUE232 of 231,551 bp, and a smaller linear plasmid pARUE113 of 112,992 bp (Figure 1). The average G + C content is 62.32%, 61.58% and 60.88%, respectively. The linear plasmid pARUE113 was already sequenced in 2007 (pAL1) [29]. The chromosome and the plasmids contain 4,575 open reading frames (ORFs), six copies of rRNA operons, 53 tRNAs and 9 pseudogenes. It shows an average coding percentage of 90%. 3382 (74%) of the protein-coding genes were assigned with a putative function and 1193 (26%) genes were annotated as hypothetical proteins. Altogether, 3625 (~78%) protein-coding genes could be assigned to Clusters of Orthologous Groups (COG) categories (Figure 2). The assignment to the COG categories is nearly identical to those of other sequenced *Arthrobacter* strains isolated from soil [18,20,21,23]. Twenty-two% (972) of all coding DNA sequences (CDSs) of *Arthrobacter* sp. Rue61a were clustered into 315 paralogous families ranging from 32 to 2 members per family.

**Figure 3:**
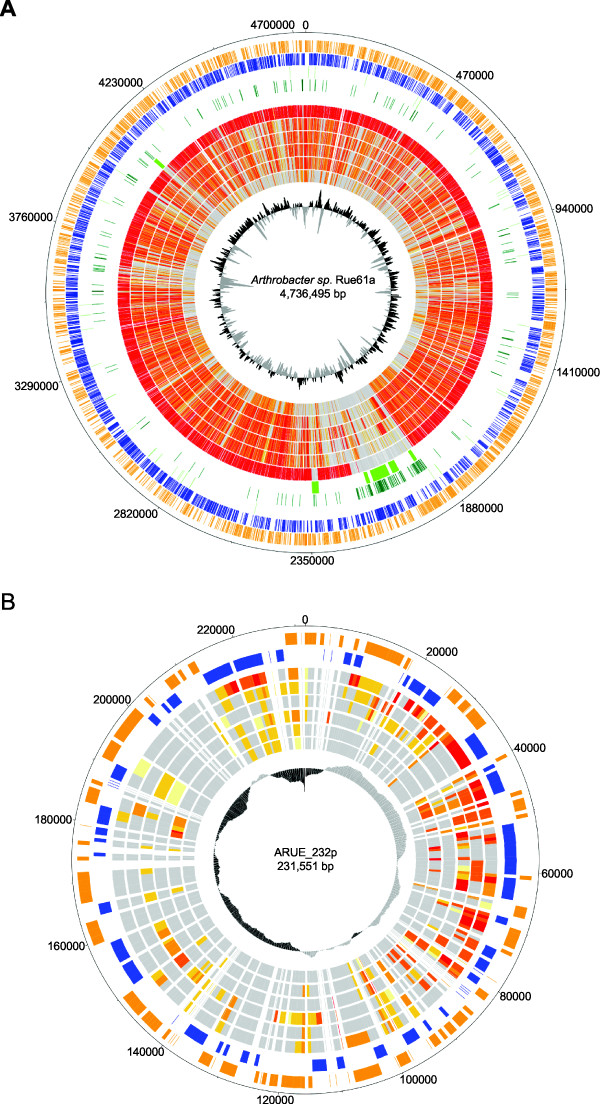
**Comparison of the chromosome and circular plasmid of *Arthrobacter *sp. Rue61a with other *Arthrobacter *species. A**: Comparison of chromosomes. Genes encoded by the leading and the lagging strand (circle 1 and 2) of the chromosome of strain Rue61a are marked in orange and blue, respectively. rRNA-clusters and tRNAs (3rd circle) are shown in pink and black, respectively. Genes of phylogenetic distribution to other phyla than actinobacteria (circle 4) and putative genomic islands (circle 5) are shown in dark green and lime green, respectively. Genome comparison using the BiBaG tool based on bidirectional blasts were done against the genomes of *A*. *aurescens* TC1, *Arthrobacter* sp. FB24, *A*. *globiformis* NBRC 12137, *A*. *chlorophenolicus* A6, *A*. *phenanthrenivorans* Sphe3, and *A*. *arilaitensis* Re117 (circles 6, 7, 8, 9, 10 and 11, respectively). The inner plot gives the G+C content. **B**: Circular plot of pARUE232. Genes encoded by the leading and the lagging strand (circle 1 and 2) of the circular plasmid pARUE232 of *Arthrobacter* sp. Rue61a are marked in orange and blue, respectively. Comparison with the plasmids of *A*. *aurescens* TC1 (circle 3) and *Arthrobacter* sp. FB24 (circle 4), the draft genome of *A*. *globiformis* NBRC 12137 (circle 5), and the plasmids of *A*. *chlorophenolicus* A6 (circle 6), *A*. *phenanthrenivorans* Sphe3 (circle 7) and *A*. *arilaitensis* Re117 (circle 8) is based on bidirectional blasts using the BiBaG tool. The inner plot gives the G+C content.

Comparison of the genome of *Arthrobacter* sp. Rue61a with the genomes of all available *Arthrobacter* strains revealed *A*. *aurescens* TC1 as the closest relative (Figure 3). The chromosomes of the two organisms show an overall similarity of approximately 86% on amino acid level, which conform to 3716 ORFs of the two chromosomes (cutoff 30% based on Needleman-Wunsch algorithm). *Arthrobacter* sp. FB24 is the next closed related organism with 2929 homologous ORFs followed by *A*. *globiformis* NBRC 12137, *A*. *chlorophenolicus* A6 and *A*. *phenanthrenivorans* Sphe3 with 2914, 2917 and 2762 ORFs, respectively. *A*. *arilaitensis* Re117 is the most distant related organism and shares 1252 homologous ORFs. In contrast, the chromosomes of all *Arthrobacter* strains share only 1014 homologous ORFs.

Analyses of the best BLAST hits (evalue cutoff e-20) of the whole genome of *Arthrobacter* sp. Rue61a revealed 303 ORFs that phylogenetically affiliated to phyla other than actinobacteria or without an assigned phylogenetic origin. These results conform to the prediction of putative genomic islands (Figure 3A) using the IslandViewer online tool [34]. Thirteen putative genomic islands were identified on the chromosome of *Arthrobacter* sp. Rue61a encoding 160 genes (see Additional file 1: Table S1).

The genome of *Arthrobacter* sp. Rue61a harbors a high number of genes associated with transport. Overall 594 putative transporters and binding proteins were identified. This represents about 13% of the CDSs of the whole genome. 512 of these transporters associated proteins are identical or closely related to these encoded in the genome of strain TC1. A predominant part of transporters-associated genes which are not related to those of TC1 (82 CDSs) are located on genomic islands (35 CDSs) or on plasmid pARUE232 (18 CDSs) (see Additional file 2: Tables S2).

### Osmoprotection systems

Soil microorganisms are exposed to frequent changes of the external osmolarity. Bacterial response to hyperosmotic stress usually involves a multiphasic adaptation, comprising a transient accumulation of potassium ions and an increase in the intracellular concentration of organic osmoprotectants by uptake and synthesis [35,36].

Secondary carriers of the Trk/Ktr family have been proposed to play an important role in the initial adjustment to an osmotic upshift. They are oligomeric complexes consisting of an ion-conducting transmembrane component associated with at least one regulatory, dinucleotide-binding component; Trk and Ktr systems are thought to differ with respect to the ion co-transported with K^+^, using H^+^ and Na^+^, respectively [37]. A putative TrkH transporter (ARUE_c34640) and its regulatory protein TrkA (ARUE_c34650) were identified in the genome of strain Rue61a; two additional homologues of TrkA are encoded on the chromosome, and the gene for another possible TrkH-type membrane protein is located on the circular plasmid pARUE232 (see Additional file 3: Table S3.1).

*Arthrobacter* sp. Rue61a has a number of uptake systems for organic osmoprotectants, as suggested by the identification of several genes predicted to code for possible proline/betaine transport systems of the major facilitator superfamily (MFS), a betaine/carnitine/choline transporter (BCCT) family protein (ARUE_c04460), which shows approximately 45% identity to the high-affinity, Na^+^-coupled glycine betaine symporter BetP of *Corynebacterium glutamicum* [PDB:3P03], and three ABC-type glycine betaine/carnitine/choline or proline/betaine transporters. Choline probably can serve as substrate for glycine betaine biosynthesis, which involves two oxidation steps. The gene product of ARUE_c04830 shares 89% sequence identity with choline oxidase of *A*. *globiformis* [PDB:2JBV], which catalyzes betaine-aldehyde formation. A putative betaine-aldehyde dehydrogenase (BetB, GbsA) is encoded by the adjacent locus.

Besides accumulating osmoprotectants from the environment, *Arthrobacter* sp. Rue61a also seems to be capable of synthesizing compatible solutes *de novo*. Analogous to the situation in mycobacteria [38] and in *Corynebacterium glutamicum*[39], three putative pathways are present for the formation of trehalose. The ARUE_c08620 and ARUE_c42120 loci were predicted to code for trehalose synthases (TreS), which catalyze a transglycosylation reaction to isomerize maltose to trehalose. However, TreS could rather be involved in trehalose degradation via maltose rather than trehalose synthesis. Genes predicted to code for trehalose-6-phosphate synthase and trehalose-6-phosphate phosphatase, comprising the OtsAB pathway for trehalose synthesis from UDP-D-glucose and α-D-glucose-6-phosphate, as well as the genes for the alternative TreYZ pathway for trehalose formation from maltodextrins are located on the chromosome of strain Rue61a (see Additional file 3: Table S3.1).

Besides glycine betaine and trehalose, ectoine and the amino acids proline and glutamate are among the most widely used compatible solutes in bacteria [36]. While strain Rue61a probably is capable of scavenging ectoine from the environment via secondary or ABC transporters, it apparently lacks the ability of *de novo* synthesis, as genes coding for homologues of the biosynthesis enzymes EctABC and ectoine hydroxylase (EctD) were not identified. In contrast, it appears to be capable of synthesizing proline from glutamate by the universal pathway encoded by *proB* (ARUE_c25190), *proA* (ARUE_c25180), and *proC* (ARUE_c34660). Genes encoding the two enzymes catalyzing glutamate synthesis are also predicted, namely, a homooligomeric NADP^+^-specific glutamate dehydrogenase, and a glutamate synthase (see Additional file 3: Table S3.1).

Whereas K^+^ uptake and accumulation of organic osmoprotectants by both uptake and synthesis contribute to adaptation of bacteria to hyperosmotic conditions, the initial response to sudden hypoosmotic shock is mediated by mechanosensitive ion channels, which act as “emergency valves” by releasing cytoplasmic solutes, relieving turgor pressure and preventing cell lysis [40]. Candidate genes for both mechanosensitive ion channels MscL and MscS were identified on the chromosome of *Arthrobacter* sp. Rue61a. All in all, the diversity of osmoprotection systems identified suggests that strain Rue61a is well adapted to cope with osmotic stress conditions.

### Endogenous generation of reactive oxygen species and protection against oxidative stress

Reactive oxygen species (ROS) are inevitable by-products of aerobic metabolism. Besides the respiratory enzymes, which are a source of superoxide, aerobic organisms have evolved a plethora of oxygenases and oxidases that use molecular oxygen as cosubstrate and electron acceptor, respectively. Among these oxidoreductases, especially the flavoenzymes have been identified as a major source of endogenous ROS [41]. Additionally, soil microorganisms may be exposed to redox-active secondary metabolites which can contribute to ROS formation [42].

*Arthrobacter* sp. Rue61a contains many genes encoding (putative) oxidases that generate H_2_O_2_ and possibly also release superoxide anion radicals as by-product. Genome analysis suggested the presence of three potential amino acid oxidases, two putative pyranose 2-oxidases, two sugar alcohol oxidases, putrescine oxidase, urate oxidase, and a number of other predicted oxidases. Moreover, there are a number of gene products that could be either oxidases or dehydrogenases, such as xanthine oxidase/dehydrogenase encoded by ARUE_c35300–35310, the sulfite oxidase family proteins, or proteins predicted to belong to the acyl-CoA dehydrogenase/oxidase superfamily (see Additional file 3: Table S3.2).

For the detoxification of H_2_O_2_, which besides causing oxidative damage of [4Fe-4S]-clusters in proteins can generate the highly reactive hydroxyl radical through the Fenton reaction [41], strain Rue61a contains three chromosomally encoded catalases, namely, a probable Mn-containing catalase and two different heme-binding catalases. Genome analysis additionally revealed a putative glutathione peroxidase, a possible peroxidase with a C(x)_2_C(x)_3_H(x)_7_G motif (TIGR01926) encoded by ARUE_c37540, another peroxidase-related protein (ARUE_c21770) and several putative peroxiredoxins (see Additional file 3: Table S3.2). These enzymes may catalyze the reduction of different organic hydroperoxides. Detoxification of superoxide is effected by a Fe/Mn-superoxide dismutase encoded by ARUE_c22440. Interestingly, the linear plasmid pARUE113 carries an additional gene (ARUE_113p00140) of an Fe/Mn-superoxide dismutase, which may be linked to quinaldine catabolism [29]. The initial reaction of the degradation pathway is catalyzed by quinaldine 4-oxidase, encoded by the genes ARUE_113p00040–00060 [28], which likely enhances internal oxidative stress when the cells are growing on quinaldine as carbon source.

Like many other bacterial genomes outside of the enterobacteria, the genome of *Arthrobacter* sp. Rue61a lacks the *soxS* regulatory gene but contains a *soxR* homologue (ARUE_c36760), which codes for a [2Fe-2S]-containing transcription factor that presumably mediates a response to redox-active metabolites. The SoxR regulon has been characterized most intensely for *Pseudomonas aeruginosa* and *Streptomyces coelicolor*, which produce the redox-cycling secondary metabolites pyocyanin and actinorhodin, respectively. For these bacteria, it has been hypothesized that SoxR has evolved to respond to their own redox-active pigments, stimulating the transcription of genes that code for proteins to process and to export these metabolites [43-45]. However, gene clusters that could code for the biosynthesis of phenazines or polyketides were not detected in the genome of *Arthrobacter* sp. Rue61a. If the strain indeed does not produce redox-active secondary metabolites, its SoxR regulon may be involved in defense against exogenous redox-active compounds.

### Acquisition of iron

Like other aerobic microorganisms, *Arthrobacter* sp. is confronted with the problem of low availability of iron ions in the oxic environment at neutral pH. In order to solubilize ferric iron, a common strategy used by bacteria is to synthesize and secrete high-affinity extracellular ferric chelators, called siderophores, and take up the Fe^3+^/siderophore complexes via active transport. Typical siderophores chelate Fe^3+^ via hydroxamate, 2-hydroxycarboxylate or catecholate ligands [46].

Interestingly, the predicted products of the ARUE_c02640–02670 gene cluster exhibit sequence similarity (28–40% identity) to RhbB, RhbE, RhbD and RhbF(/C) of *Sinorhizobium meliloti* which catalyze schizokinen formation from 2,4-diaminobutyrate, acetyl-CoA and citrate [47], suggesting that *Arthrobacter* sp. Rue61a may produce a schizokinen-like siderophore. Schizokinen from *Bacillus megaterium* and arthrobactin from *Arthrobacter pascens* are dihydroxamates, composed of citric acid symmetrically substituted via amide linkages to two residues of 1-amino-3-(*N*-acetylhydroxyamino)propane and 1-amino-5-(*N*-acetylhydroxyamino)pentane, respectively [48,49]. Schizokinen is also the direct biosynthetic precursor of rhizobactin-1021 [50], a siderophore produced by *S. meliloti* 1021.

The proteins encoded by the ARUE_c39970–40000 gene cluster belong to the CeuD, CeuC, CeuB and CeuA families and thus may represent a system that transports Fe^3+^/catecholate-type siderophore complexes. Apart from a predicted isochorismate synthase (ARUE_c32560), genes for the biosynthesis of the catecholate siderophore precursor 2,3-dihydroxybenzoate and gene clusters for non-ribosomal siderophore peptide synthetase components were not detected, suggesting that the Ceu-like proteins contribute to acquisition of xenosiderophore/Fe^3+^ complexes (i.e., siderophores produced by other bacteria). At large, strain Rue61a forms five ABC transporters for the uptake of Fe^3+^/siderophore complexes (see Additional file 3: Table S3.3).

The gene products of the ARUE_c07040, ARUE_c07050 and ARUE_c07060 loci show about 49%, 59% and 66% identity to the SfuC, SfuB and SfuA proteins, respectively, of *Serratia marcescens*[51]. In contrast to siderophore-mediated iron uptake processes, which involve internalization of the entire Fe^3+^/siderophore complexes, the SfuABC (FbpABC) system binds and transports free ferric ions. In pathogenic bacteria like *Neisseria gonorrhoeae*, *N*. *meningitidis* and *Haemophilus influenzae*, the FbpABC system is required for ferric iron acquisition from the host's iron binding proteins transferrin and lactoferrin, and it also contributes to the utilization of some xenosiderophores as iron sources [52-54]. Sequence similarities of the ARUE_c07040–07060 proteins to FbpABC of *H*. *influenzae* and *N*. *gonorrhoeae* are lower than towards SfuABC (34%–42% identical amino acids), however, the amino acid ligands, which in *H*. *influenzae* FbpA coordinate the Fe^3+^ ion (H9, E57, Y195 and Y196; [PDB:1MRP and PDB:2O6A]) [55], are conserved in the ARUE_c07060 protein. Homologous proteins can be identified in other *Arthrobacter* spp. and in rhodococci, suggesting that these Gram-positive soil bacteria may be able to scavenge ferric ions by means of a an FbpA-like extracellular Fe^3+^-binding protein and an Fe^3+^-specific ABC transporter. Two *fur* homologs coding for regulators that sense Fe^2+^ or other divalent metal ions and control metal ion homeostasis were identified in the genome (see Additional file 3: Table S3.3).

### Response to heavy metal ions

Metal ions are usually toxic if present in excess, mainly due to their reactivity with thiol compounds and the thiolate and imidazolium groups of cysteine and histidine residues, respectively. Some divalent metal ions such as Cd^2+^ tend to substitute physiologically essential metal ions that act as enzyme cofactors or as structural constituents of proteins.

Co^2+^, Ni^2+^, Cu^2+^ and Zn^2+^ ions inhibited growth of *Arthrobacter* sp. strain Rue61a at approximately 2 mM, whereas Cd^2+^, a metal ion without known biological function, was effective already at μM concentrations (Table 1). Uptake systems for macronutrients like Mg^2+^, sulfate and phosphate generally exhibit a broad specificity, consequently the uptake of toxic metal ions and metal oxyanions cannot be down-regulated on the level of transport activity [56]. Energy-dependent efflux is therefore the primary mechanism for the detoxification of metal ions [57]. The CzcD-like proteins encoded by ARUE_c03850 and ARUE_232p00430 belong to the cation diffusion facilitator (CDF) family of antiporters [57] and might contribute to the observed moderate degree of Zn^2+^ and Co^2+^ tolerance of strain Rue61a. The gene ARUE_232p00330 codes for a putative antiporter that is related to the NreB protein of plasmid pTOM9 of *Alcaligenes xylosoxidans* strain 31A (55% identity), but since it lacks the characteristic histidine-rich carboxy terminus [57], its possible role in nickel efflux is uncertain. A putative CopC-related protein, also encoded on the linear plasmid, presumably contributes to copper homeostasis by sequestration of copper ions (see Additional file 3: Table S3.4).

**Table 1:** **Minimal inhibitory concentrations (MIC) of metal salts for *Arthrobacter *sp. Rue61a**

**MIC (mM)**									
K_2_CrO_4_	CoCl_2_	NiSO_4_	CuSO_4_	ZnSO_4_	NaAsO_2_	Na_2_HAsO_4_	CdSO_4_	HgCl_2_	Pb(NO_3_)_2_
5.0	2.0	2.0	2.5	2.0	0.2	400	0.006	0.012	5.0

The strain was grown in nutrient broth (½ LB), and growth was recorded after 48 h.

**Figure 4:**
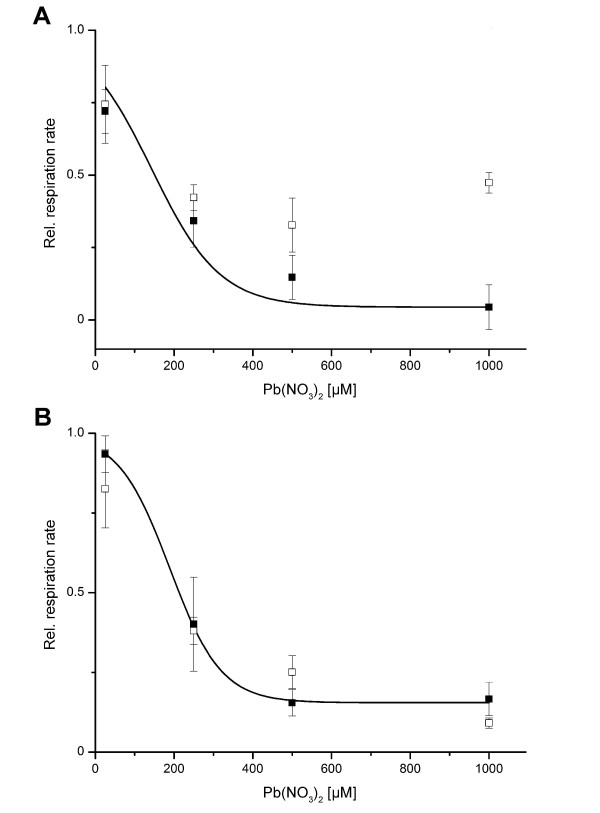
**Relative respiration rates of *Arthrobacter *sp. Rue61a and *A*. *aurescens *TC1 in response to Pb**^**2+**^**. ***Arthrobacter* sp. Rue61a (**A**) and *A. aurescens* TC1 (**B**) were cultivated in LB medium in the presence and absence of 10 μM Pb(NO_3_)_2_. Cells were resuspended in 10 mM MES buffer, pH 6.5, and respiration rates of cells pre-grown in the presence of Pb(NO_3_)_2_ (empty squares) and of cells grown in the absence of Pb^2+^ (filled squares) were measured at different concentrations of Pb(NO_3_)_2_. The relative respiration rate is the ratio of the ΔO_2_ after and prior to Pb(NO_3_)_2_ injection. Data represent mean values ±SD (standard deviations) from three independent experiments. The continuous lines represent fits of a dose–response equation to the data of the LB-grown cells.

A number of important efflux systems for heavy metal cations are P-type ATPases. CopA is a Cu^+^/Ag^+^-ATPase [58,59], the ZntA protein mediates resistance to Zn^2+^, Cd^2+^ and Pb^2+^ and has low activities with Ni^2+^, Co^2+^ and Cu^2+^[59], CadA is a Cd^2+^ transporter also active with Pb^2+^, and PbrA mediates Pb^2+^ resistance [59,60]. The P-type ATPase encoded by ARUE_c42400, which includes an N-terminal heavy metal-associated HMA domain (cd00371) with the typical CxxC motif, is related to CopA and thus is a likely candidate for a transporter that mediates Cu^+^/Ag^+^ efflux. The adjacent gene codes for a putative metal-binding chaperone that delivers the metal ion to the transporter. Another putative metal-transporting P-type ATPase is encoded by the ARUE_c19540 locus, and the protein encoded by ARUE_232p00780 shows similarity to copper-translocating ATPases. The circular plasmid comprises two additional genes, ARUE_232p00560 and ARUE_232p00710, of predicted Cd^2+^/Zn^2+^/Pb^2+^-translocating P-type ATPases (see Additional file 3: Table S3.4). The deduced proteins have an N-terminal CxxD motif and additional conserved glutamate residues in their N-terminal region, reminiscent of the metal-associated motif of PbrA [60]. Homologues of these genes can be identified in the genome of *A*. *aurescens* TC1 (AAur_pTC20031, AAur_pTC10188). We observed that growth of both *Arthrobacter* sp. Rue61a and *A*. *aurescens* TC1 in half-concentrated LB medium was inhibited by 5 mM Pb(NO_3_)_2_ (Table 1). This is comparable to the MICs of 5–10 mM Pb(NO_3_)_2_, determined (in TY broth) for *Arthrobacter* strains isolated from lead-zinc mine tailings [6]. However, since lead precipitates with various anions and forms complexes with many organic compounds, its toxicity towards strains Rue61a and TC1 was additionally assessed be measuring its effect on the respiration rate of resting cells suspended in buffer [61]. The IC_50_ values (concentration resulting in a 50% decrease in respiration rate) for LB-grown cells of strains Rue61a and TC1, deduced from dose–response curves (Figure 4), were approximately 143 μM and 189 μM Pb(NO_3_)_2_, respectively. Considering that IC_50_ values of 37 μM and 20 μM were reported for Pb^2+^-induced and non-induced resting cells of *Arthrobacter* sp. JS7, an isolate from a Pb-contaminated industrial site [61], the resistance levels of *Arthrobacter* strain Rue61a and *A*. *aurescens* TC1 are remarkably high.

A putative mercuric reductase gene *merA*, flanked by a gene coding for a MerR family transcriptional regulator, is represented by ARUE_232p00800. The gene product shares 59% sequence identity with MerA of the Hg-resistant *Streptomyces* sp. strain CHR28. However, HgCl_2_ retarded growth of strain Rue61a already at 3 μM, and complete inhibition occurred at 12 μM (Table 1), indicating high sensitivity towards Hg^2+^ ions.

Chromate enters bacterial cells via the sulfate uptake system [57]. Whereas *Arthrobacter* sp. strain FB24 exhibits high-level chromate resistance, surviving up to 200 mM potassium chromate, resistance levels of other *Arthrobacter* species were in the range of 2 to 48 mM [23]. Growth of strain Rue61a was unaffected by 2.5 mM but inhibited by 5 mM chromate (Table 1), suggesting only moderate tolerance. A widespread bacterial resistance mechanism is chromate efflux, mediated by the ChrA transporter [23,57], but a ChrA ortholog was not identified in the genome of strain Rue61a. However, another mechanism of chromate resistance that seems to be wide-spread in bacteria is based on reduction to Cr(III), which at physiological pH forms oxides and hydroxides that are poorly soluble and thus less bioavailable than the Cr(VI) anions [62]. Chromate reduction was reported for *A*. *crystallopoietes* ES32 and *Arthrobacter* sp. strain CR47 [63,64], but the genetic basis of the reductase activity was not analyzed. Interestingly, the NAD(P)H-dependent FMN reductase encoded by ARUE_c41610 is related to chromate reductases ChrR of *Pseudomonas putida* and YieF of *Escherichia coli* K12 [65], sharing amino acid motifs that are highly conserved within ChrR homologs. The ability to reduce chromate also seems to be a secondary function of several NAD(P)H:flavin oxidoreductases, such as some nitroreductases and ferric reductase [62]. Since candidate genes that may confer such secondary chromate reductase activities were identified on the chromosome (see Additional file 3: Table S3.4), strain Rue61a presumably is able to detoxify Cr(VI) by reduction.

**Figure 5:**
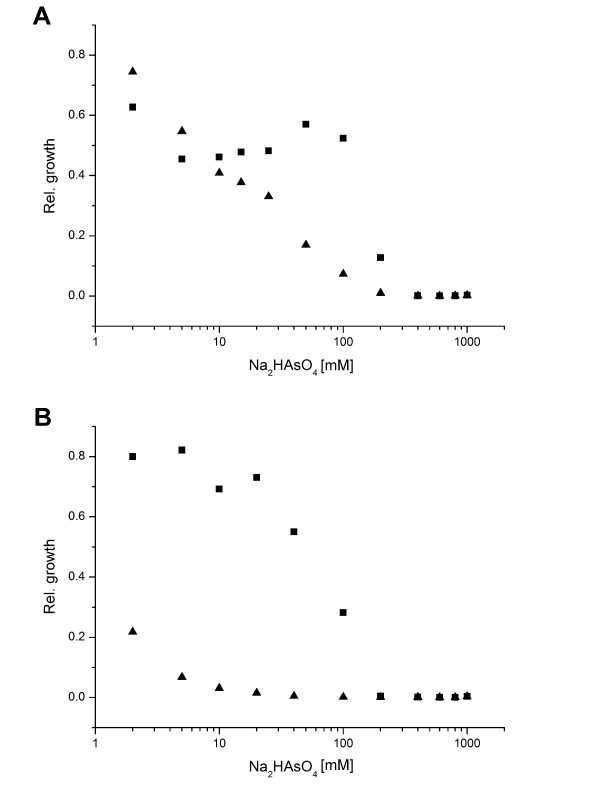
**Relative growth of *Arthrobacter *sp. Rue61a and *A*. *aurescens *TC1 in the presence of arsenate. ***Arthrobacter* sp. Rue61a (**A**) and *A*. *aurescens* TC1 (**B**) were grown in half-concentrated LB medium, supplemented with arsenate at the concentrations indicated. The turbidity of the cultures was measured after 24 h and 48 h with a Klett colorimeter, and the turbidity of the culture without arsenate was normalized to 1. Triangles and squares indicate relative growth of the arsenate-supplemented cultures after 24 h and 48 h, respectively

In oxic environments, arsenic predominates as arsenate, which is taken up by phosphate transporters and exerts toxicity by “uncoupling” oxidative phosphorylation due to formation of rapidly hydrolyzable arsenyl-ADP [66]. *Arthrobacter* sp. strain Rue61a was able to grow in the presence of up to 200 mM arsenate, albeit with prolonged lag phases at concentrations >10 mM arsenate (see Figure 5A for relative growth, as compared to a culture without arsenate, after 24 and 48 h). Full growth inhibition was observed at 400 mM arsenate (Table 1). Aerobic microorganisms usually detoxify arsenate by reduction to arsenite, catalyzed by the cytoplasmic arsenate reductase ArsC, and subsequent arsenite extrusion. Arsenite formation by ArsC must be closely coupled to arsenite efflux [67], because arsenite due to its thiolate-modifying activity is much more toxic than arsenate. Growth of *Arthrobacter* sp. strain Rue61a was inhibited by 0.2 mM arsenite (Table 1). An ArsB-like anion permease and a putative ACR3-type arsenite efflux pump are encoded by ARUE_c26820 and ARUE_232p00610, respectively.

A chromosomal gene cluster comprising ARUE_c37620–37670 contains three *arsC* paralogs coding for putative arsenate reductases. Interestingly, the genome of *A*. *aurescens* TC1 contains a total of five putative arsenate reductase genes. However, the observed MIC of 200 mM and the prolonged lag phases of cultures of strain TC1 grown in the presence of 2 – 100 mM arsenate (Figure 5B) indicate that this strain is more sensitive than strain Rue61a, suggesting that the expression levels or the catalytic efficiencies of the arsenate detoxifying proteins rather than the number of putative detoxification genes govern the physiological response. Nevertheless, both strains are highly resistant to arsenate. *Geobacillus kaustophilus* HTA426 [68], *Bacillus cereus* strain AG27 [69], and *B*. *subtilis* strain B-1232 [70], for example, exhibited MICs of 80 mM, 40 mM, and 110 mM of arsenate, respectively. Aerobic growth of eleven bacterial isolates from arsenic-contaminated groundwater was inhibited by arsenate concentrations in the range of 100–200 mM [71].

### Response to antibiotics

A plethora of antibiotics is produced by soil microorganisms. To counteract their effect, antibiotic producers as well as non-producing soil-dwelling bacteria have evolved various resistance factors, e.g., efflux transporters, or enzymes catalyzing derivatization or cleavage of the antibiotic [72].

Agar disk diffusion tests indicated that *Arthrobacter* sp. Rue61a is sensitive towards ampicillin (2 μg/disk), lincomycin (5 μg/disk), the fluoroquinolone ciprofloxacin (5 μg/disk), and the macrolide antibiotics erythromycin (0.1 μg/disk) and tylosin (2 μg/disk). These results contrasted with the detection of a number of genes encoding MFS transporters of the EmrB/QacA subfamily, several genes coding for ABC transporters predicted to be involved in multidrug export (see Additional file 2: Tables S2), and genes of two small multidrug resistance proteins (cl00910), putative rRNA methyltransferases, a putative penicillin acylase, and several predicted β-lactamases. Zones of inhibition by puromycin were observed at 10 μg/disk, suggesting that efficient efflux as mediated by a Pur8-like transporter does not occur [73]. In contrast, inhibition by the peptide antibiotic bacitracin occurred only at >100 μg/disk, indicating resistance. Several ABC-type systems are candidates for possible bacitracin efflux transporters (see Additional file 3: Table S3.5). The ATPase protein encoded by ARUE_c26560 shows 38% sequence identity to BceA, a component of the bacitracin transporter BceAB of *Bacillus subtilis* strain 168. The *bceAB* genes of *B*. *subtilis* are located adjacent to *bcrRS* which code for an associated two-component regulatory system [74]. The *bceA* homologue ARUE_c26560, and ARUE_c26570 encoding a putative permease component, also co-localize with genes coding for a predicted sensor histidine kinase and response regulator. Co-localization of ABC transporter genes and genes of a two-component regulatory system is observed for two other gene clusters on the chromosome. The product of the ARUE_c43050 gene is distantly related to the BcrA protein of the bacitracin transporter of *B*. *licheniformis* (about 37% sequence identity). Two other ABC-type transporters (see Additional file 3: Table S3.5) show weak similarity of their ATPases with those of bacitracin transporters, but these systems lack co-localized sensor kinase and response regulator genes.

### Biodegradative capabilities

#### Aromatic and N-heterocyclic compounds

*Arthrobacter* sp. Rue61a was originally isolated based on its ability to grow on quinaldine as sole source of carbon and energy. The enzymes of the “upper” part of the degradation pathway, which are encoded by ARUE_113p00040–00090 (see Additional file 4: Figure S1), have been characterized previously [28,30,31]. The intermediates 1*H*-4-oxoquinaldine and 1*H*-3-hydroxy-4-oxoquinaldine of the pathway are structural analogs of the quorum sensing signaling molecules HHQ (2-heptyl-4(1*H*)-quinolone) and PQS (2-heptyl-3-hydroxy-4(1*H*)-quinolone) of *Pseudomonas aeruginosa*. The enzymes 1*H*-4-oxoquinaldine 3-monooxygenase and 1*H*-3-hydroxy-4-oxoquinaldine 2,4-dioxygenase from strain Rue61a are indeed capable of catalyzing the hydroxylation of HHQ to PQS (unpublished data) and the cleavage of PQS [75], respectively. However, the catalytic activities of the monooxygenase and the 2,4-dioxygenase towards these signaling molecules are much lower than towards the physiological substrates, supporting the notion that the proteins have not evolved to act as a quorum quenching enzymes, but have a catabolic role.

Anthranilate, the product of the “upper pathway” of quinaldine degradation by strain Rue61a, has been proposed to be degraded via CoA-thioester intermediates; an anthranilate CoA ligase and a putative 2-aminobenzoyl-CoA monooxygenase/reductase to form 2-amino-5-oxocyclohex-1-enecarboxyl-CoA are encoded by ARUE_113p00220 and ARUE_113p00230, respectively [29] (see Additional file 3: Table S3.6 and Additional file 4: Figure S1). Since the pARUE113-deficient mutant of strain Rue61a is still able to grow on anthranilate, paralogs of these “lower pathway” genes must be present. Probable candidate genes are ARUE_c41730 and ARUE_c41720, respectively; the gene product of the latter shares 50% sequence identity with 2-aminobenzoyl-CoA monooxygenase/reductase of *Azoarcus evansii*[76]. Heterologous expression of the ARUE_113p00220 and ARUE_c41730 genes, inserted in the pET22b(+) expression vector, in *E*. *coli* resulted in anthranilate-CoA ligase activities of approximately 0.35 U (mg protein)^–1^ in the cell extract supernatants, while extracts of *E*. *coli* cells harboring the empty vector did not show enzymatic activity towards anthranilate, confirming that the two genes code for anthranilate CoA ligases.

To further validate the proposed degradation pathway, we performed LC-MS analysis of cell extracts of *Arthrobacter* sp. Rue61a, cultivated on different carbon sources (see Additional file 5: Table S4). In extracts of anthranilate-grown cells, a total of 18 different CoA esters were identified, including anthraniloyl-CoA. Besides, CoA esters of acids with the empirical formula C_7_H_7_NO_3_ and C_7_H_8_O_4_ were exclusively detected in extracts of anthranilate-grown cells. The CoA thioester of the C_7_H_7_NO_3_ acid presumably corresponds to hydroxyanthraniloyl-CoA. Interestingly, 5-hydroxyanthraniloyl-CoA was reported to be formed in the reaction catalyzed by 2-aminobenzoyl-CoA monooxygenase/reductase under NADH-limiting conditions [77]. The CoA thioester of C_7_H_8_O_4_ might correspond to cyclohexanedione-carboxyl-CoA. Cyclohexanedione was previously observed as product of the hydrolytic deamination and acidic decarboxylation of 2-amino-5-oxocyclohex-1-enecarboxyl-CoA, which is the main product of the enzymatic reaction when NADH is not limiting [77]. 2-Amino-5-oxocyclohex-1-enecarboxyl-CoA is unstable at pH <5 [77]; consistently, it could not be detected in the cell extracts.

Unexpectedly, anthraniloyl-CoA was not detected in extracts of 1 *H*-4-oxoquinaldine-grown cells (see Additional file 5: Table S4). However, the extracts contained a metabolite with the molecular formula of phenylacetyl-CoA, tentatively suggesting that a phenylacetate CoA ligase (PaaK) is upregulated under these growth conditions. If so, induction of *paaK* (ARUE_c33810) expression could be due to cross-regulation by transcriptional regulators encoded by ARUE_113p00160 and ARUE_113p00240, which both are related to the PaaX repressor of the phenylacetate catabolon, and which are involved in transcriptional control of quinaldine degradation (to be published elsewhere).

Since catechol was previously observed to occur during quinaldine degradation [26], we had discussed the presence of an alternative pathway of anthranilate degradation via catechol [29]. However, searching the genome for possible anthranilate 1,2-dioxygenase genes was inconclusive. Unexpectedly, catechol itself did not support growth, but was subject to slow cometabolic conversion by resting cells, presumably due to low activity of protocatechuate 3,4-dioxygenase towards catechol (Table 2). The metabolite which accumulated in the culture media showed an intense absorption band at 258 nm, suggesting formation of *ciscis*-muconate [78]. Since neither catechol nor monohydroxy-substituted anthranilates supported growth of strain Rue61a (Table 2), the metabolism of anthranilate appears to exclusively follow a CoA-thioester pathway.

**Table 2:** **Substrates converted by *Arthrobacter *sp. Rue61a**

**Carbon source, concentration**	**Prediction from genome**	**Growth in mineral medium/comments**
***Aromatic compounds***		
Benzoate, 2 mM	incomplete	–
2-Nitrobenzoate, 2 mM	absent	–
Anthranilate (2-aminobenzoate), 2 mM	present, CoA thioester pathway	+
3-Hydroxyanthranilate (2-amino-3-hydroxybenzoate), 2 mM	absent	–/cometabolic conversion
5-Hydroxyanthranilate (2-amino-5-hydroxybenzoate), 2 mM	n.s.	–/cometabolic conversion
Salicylate (2-hydroxybenzoate), 2 mM	absent	–
3-Hydroxybenzoate, 2 mM	n.s.	–
4-Hydroxybenzoate, 2 mM	present, via protocatechuate	+
Gentisate (2,5-dihydroxybenzoate), 2 mM	n.s.	–
Protocatechuate (3,4-dihydroxybenzoate), 2 mM	present, *ortho* pathway	+
Vanillate (4-hydroxy-3-methoxybenzoate), 2 mM	present	+
Phenylethylamine, 2 mM	incomplete	–
Phenylacetate, 2 mM	incomplete	–/cometabolic conversion
4-Hydroxyphenylacetate, 2 mM	present, via homoprotocatechuate	+
Homoprotocatechuate (3,4-dihydroxyphenylacetate), 2 mM	present, *meta* pathway	+
Tyrosine, 2 mM	present, via 4-hydroxyphenylacetate and homoprotocatechuate	+
4-Hydroxymandelate [2-hydroxy-2-(4-hydroxyphenyl)acetate], 2 mM	incomplete	–
Phenol, 1 mM	incomplete; possible monooxygenation	–/no hydroxylation to catechol
Catechol, 1 mM	incomplete	–/cometabolic conversion to *cis*,*cis*-muconate within 1–2 days (cells pre-grown with protocatechuate) or 4–5 days (cells grown on other C sources)*
Styrene (ethenylbenzene), 1 mM	incomplete	–/cometabolic conversion
Biphenyl, 2 mM	incomplete	–
4-Phenoxybenzoate, 1 mM	n.s.	–
* by resting cells (OD_600 nm_ ~20) pre-grown in MM supplemented with glucose as carbon source (0.5%), or with glucose plus 1 mM or 2 mM of either benzoate, anthranilate, biphenyl, homoprotocatechuate, protocatechuate, or catechol.		
***N*-*Heterocyclic compounds***		
Hypoxanthine and xanthine, 2 mM	present	+
Quinaldine (2-methylquinoline), 2 mM	present	+
1*H*-4-Oxoquinaldine, 1 mM	present	+
1*H*-3-Hydroxy-4-oxoquinaldine, 1 mM	present	+
***Sugars (and *-*derivatives)***		
Glucose, 0.25% and 0.5%	present	+
Fructose, 0.5%	present	+
Galactose, 0.5%	present	+
L-Arabinose, 0.5%	present	+
Mannose, 0.5%	present	+
Ribose, 0.5%	present	+
Rhamnose, 0.5%	incomplete	+
Xylose, 0.5%	present	+
Lactose, 0.25%	present	+
Sucrose, 0.25%	present	+
Trehalose, 0.25%	possibly via maltose	+
Maltose, 0.25%	n.s.	+
D-Glucosamine, 0.5%	present	+
N-Acetyl-β-D-glucosamine, 0.5%	present	+
***Alcohols and carboxylic acids***		
Ethanol (0.5% and 1%)	present	+
Glycerol (1%)	present	+
2-Chloroacetate, 1 mM, 3 mM, 10 mM	n.s.	–
Acetate, 1 mM, 3 mM	present	+
Glycolate (2-hydroxyethanoic acid), 10 mM	absent	–
Glyoxylate, 10 mM	present	+
Pyruvate, 40 mM	present	+
Glutarate, 10 mM	absent	–
D-Gluconate, 0.5%	present	+
***Lipids***		
Triacylglycerides: Tributyrin	absent	–/no zones of clearing on tributyrin plates
Monoacylglycerides: 1-Oleyl-*rac*-glycerol, 2 mM	present	+
***Natural polymers***		
Starch	present	+/growth and zones of clearing on starch plates
Pectin	n.s.	–
Lichenin	n.s.	–
Carboxymethylcellulose	n.s.	–
Chitin	n.s.	–
Protein	present	+/growth and zones of clearing on skim milk plates
***Amines*, *amides and related compounds***		
Choline (*N*,*N*,*N*-trimethylethanolammonium chloride), 0.2%	via glycine betaine	+
Glycine betaine (2-trimethylammonioacetate), 0.5%	incomplete	+
Creatinine (2-amino-1-methyl-5*H*-imidazol-4-one), 0.2%	absent	–
Creatine [2-(1-methylcarbamimidamido)acetate], 0.2%	present, via sarcosine	+
Sarcosine [2-(methylamino)acetic acid], 0.5%	present	+
Putrescine (butane-1,4-diamine), 5 mM	present, via 4-aminobutyrate	+
Agmatine [N-(4-aminobutyl)guanidine sulfate], 15 mM	incomplete, via putrescine	+
Allantoin [(2,5-dioxo-4-imidazolidinyl)urea], 0.2%	incomplete	+
		
Taurine (2-aminoethanesulfonic acid), 10 mM	incomplete	–
**Nitrogen sources**	**Prediction from genome**	**Growth in mineral medium with glucose**
Urea, 0.2%	present	+
Allantoin [(2,5-dioxo-4-imidazolidinyl)urea], 0.02%, 0.2%	incomplete	+
Creatine [2-(methylguanidino)ethanoic acid], 0.2%	present	+
Putrescine (butane-1,4-diamine), 5 mM	present	+
Agmatine [N-(4-aminobutyl)guanidine sulfate], 5 mM	incomplete	+
Taurine (2-aminoethanesulfonic acid), 5 mM	absent	–
**Sulfur source**	**Prediction from genome**	**Growth in mineral medium with glucose**
Taurine (2-aminoethanesulfonic acid), 1 mM	sulfite formation by taurine dioxygenase	+

n.s., not specified.

Growth of *Arthrobacter* sp. strain Rue61a on glucose-containing mineral medium supplemented with benzoate resulted in the presence of benzoyl-CoA in cell extracts (see Additional file 5: Table S4), which might be formed by the ARUE_c41730 and/or ARUE_113p00220 proteins, however, benzoate did not support growth. The strain also does not grow on salicylate, 3-hydroxybenzoate, or gentisate. In contrast, 4-hydroxybenzoate was readily utilized (Table 2), presumably via hydroxylation to protocatechuate by PobA monooxygenase and subsequent mineralization via the *ortho* ring-cleavage pathway encoded by the *pca* gene cluster. Vanillate most probably is converted to protocatechuate by a vanillate O-demethylase prior to mineralization via *ortho* cleavage (see Additional file 4: Figure S2). In contrast, a *meta* cleavage pathway can be proposed for the degradation of 4-hydroxyphenylacetate and homoprotocatechuate (see Additional file 3: Table S3.6 and Additional file 4: Figure S3). Interestingly, 4-hydroxyphenylacetate is an intermediate of a tyrosine degradation pathway found in several Gram-positive bacteria [79,80]. While tyrosine and 4-hydroxyphenylacetate supported growth of strain Rue61a (Table 2), phenylacetate did not, but underwent rapid cometabolic conversion to an unknown metabolite. A gene cluster comprising potential *paa* genes is formed by ARUE_c33590–33810. The deduced gene products of ARUE_c33590–33640 share between 68% and 87% sequence identity with the PaaBKJIHG proteins of *A*. *oxydans* CECT386 [81], which presumably represent an oxepin-CoA-forming ring 1,2-epoxyphenylacetyl-CoA isomerase (PaaB; *E*. *coli*: PaaG), and the five components of a ring 1,2-phenylacetyl-CoA epoxidase (PaaGHIJK; *E*. *coli*: PaaABCDE) [82]. The putative phenylacetate CoA ligase (*E*. *coli*: PaaK) encoded by ARUE_c33810 shows 86% identity to PaaF of *A*. *oxydans*, and ARUE_c33800 is presumed to code for a thioesterase (69% identity to PaaD of *A*. *oxydans*; *E*. *coli*: PaaI). The apparently incomplete set of *paa* genes is consistent with the finding that phenylacetate is transformed without being degraded. Styrene also was not utilized, but cometabolically converted (Table 2), possibly by the gene products of ARUE_c02440 and ARUE_c02450, which show 53% and 67% identity to the self-sufficient styrene monooxygenase StyA2B and the associated StyA1 protein, respectively, of *Rhodococcus opacus* strain 1CP [83]. Taken together, strain Rue61a apparently has a quite narrow set of aromatic degradation pathways, which mainly enable the utilization of 4-hydroxy-substituted aromatic carboxylic acids. Compounds like vanillate and 4-hydroxybenzoate are characteristic products of lignin depolymerization, suggesting that strain Rue61a has adapted to utilize the low molecular weight aromatic compounds produced by ligninolytic microorganisms.

#### Carbohydrates and carboxylic acids

*Arthrobacter* sp. Rue61a is able to utilize a number of monosaccharides, the disaccharides sucrose, lactose, trehalose and maltose, as well as glucosamine and *N*-acetylglucosamine. Trehalose probably is isomerized to maltose by TreS. A gene for maltose phosphorylase could not be identified, however, a putative *alpha*-glucan phosphorylase gene is located closely to one of the *treS* genes. Starch is utilized, consistent with the prediction of a secreted alpha-amylase (ARUE_c02210), but other polysaccharides tested were not hydrolyzed (Table 2). The central pathways of carbohydrate metabolism, glycolysis, pentose phosphate cycle, tricarboxylic acid cycle and gluconeogenesis, were identified in the genome. The gene encoding the anaplerotic enzyme phosphoenolpyruvate carboxylase is also present (ARUE_c07170). Genes coding for isocitrate lyase and malate synthase, the key enzymes of the anaplerotic glyoxylate cycle, were also identified. Consistent with the predicted glyoxylate cycle proteins, acetate and glyoxylate were readily used as carbon sources. However, utilization of glyoxylate may proceed mainly via the D-glycerate pathway (see Additional file 4: Figure S7). 2-Chloroacetate as well as glycolate, which would result from hydrolytic dechlorination of chloroacetate, did not support growth (Table 2).

#### Proteins, lipids, and alcohols

*Arthrobacter* strain Rue61a grows on skim milk agar plates and produces zones of clearing around the colonies, indicating extracellular proteolytic activity. Several genes for putative secreted proteases are present in the genome. Utilization or cometabolic hydrolysis of the triacylglyceride tributyrin was not observed, but the monoacylglyceride 1-oleyl-*rac*-glycerol supported growth of *Arthrobacter* sp. Rue61a. The genome contains several genes coding for putative lipases/esterases of the α/β-hydrolase fold-superfamily or the SGNH-superfamily, however, signal peptides were not predicted for these proteins. Growth on glycerol probably involves the activity of glycerol kinase (GlpK) and glycerol-3-phosphate dehydrogenase (GlpD) encoded by ARUE_c24020 and ARUE_c24040, respectively, to form dihydroxyacetone phosphate. The ARUE_c24030 gene within this cluster codes for a putative glycerol uptake facilitator protein. The ARUE_c30140 locus codes for another GlpD protein. Whereas the glycerol-3-phosphate dehydrogenase GlpD is an NAD-independent flavoenzyme that passes electrons to the respiratory chain, GpsA-type glycerol-3-phosphate dehydrogenase, presumably represented by ARUE_c26370, depends on NAD^+^.

The genome of *Arthrobacter* strain Rue61a contains several genes of putative alcohol dehydrogenases and aldehyde dehydrogenases/oxidases, but since the amino acid sequence of the protein encoded by ARUE_c32130 shares 74% identity with acetaldehyde dehydrogenase II of *Cupriavidus necator* (formerly, *Ralstonia eutropha*) H16 [84], ethanol utilization by strain Rue61a likely involves this protein.

#### Amines and other nitrogen compounds

Among the amine compounds tested as sources of carbon and energy and as nitrogen sources, creatinine was not utilized, but creatine and sarcosine supported growth of *Arthrobacter* sp. Rue61a (Table 2). Creatine degradation involves hydrolysis to sarcosine (*N*-methylglycine) and urea by creatinase, followed by oxidation of sarcosine to formaldehyde and glycine. The gene arrangement of ARUE_c39610–39670 corresponds to that of gene clusters of *Corynebacterium* sp. U-96 and *Arthrobacter* spp. coding for serine hydroxymethyltransferase (*glyA*), tetrameric sarcosine oxidase (*soxBDAG*), serine dehydratase (*sdaA*), and 10-formyltetrahydrofolate deformylase (*purU*) [85,86], suggesting that sarcosine catabolism in *Arthrobacter* sp. strain Rue61a proceeds via glycine and serine to pyruvate (see Additional file 4: Figure S4). In *Corynebacterium* sp., the tetrameric sarcosine oxidase is the catabolic enzyme that is induced during growth on sarcosine [87].

*Arthrobacter* sp. strain Rue61a also grows on choline. As mentioned above, choline presumably is a source for synthesis of the osmoprotectant glycine betaine, but its utilization as carbon source probably also proceeds via glycine betaine (see Additional file 4: Figure S4). The protein encoded by ARUE_c04530, which shows 87% identity to *N*,*N*-dimethylglycine oxidase of *Arthrobacter globiformis* [PDB:1PJ7], is a likely candidate for the enzyme catalyzing *N*,*N*-dimethylglycine conversion to sarcosine.

Agmatine seems to be utilized via the agmatine deiminase (AguA) pathway, which involves hydrolysis of agmatine to ammonia and carbamoylputrescine by AguA and another hydrolytic step to putrescine, catalyzed by *N*-carbamoylputrescine amidohydrolase AguB. Putrescine degradation follows oxidative deamination to 4-aminobutanal, presumably catalyzed by the ARUE_c00400 protein, which shares 76% identity with putrescine oxidase of *Rhodococcus erythropolis* [PDB:2YG3]. Oxidation of 4-aminobutanal by γ-aminobutyraldehyde dehydrogenase produces 4-aminobutyrate, which in a transamination reaction is converted to succinic semialdehyde. Oxidation by succinate semialdehyde dehydrogenase finally yields succinic (see Additional file 4: Figure S5).

Hypoxanthine and xanthine utilization by *Arthobacter* sp. strain Rue61a is initiated by oxidation to urate. The reaction is catalyzed by xanthine dehydrogenase or -oxidase, a molybdenum hydroxylase encoded by ARUE_c35300–35310. Urate degradation probably involves urate oxidase and 5-hydroxyisourate hydrolase. The next step, catalyzed by OHCU decarboxylase, produces (*S*)-allantoin (see Additional file 4: Figure S6). Allantoin indeed supports growth of *Arthrobacter* sp. Rue61a and also can serve as sole nitrogen source. Its degradation likely occurs via glyoxylate (see Additional file 4: Figure S7). A putative allantoinase gene is located adjacent to the genes coding for glyoxylate utilization via the glycerate pathway (ARUE_c36330–36350), however, candidate genes for allantoate conversion to urea and glyoxylate, or to ammonia, carbon dioxide, urea and glyoxylate, were not obvious. Two pathways seem to be present for the degradation of urea, namely, direct cleavage by urease (*ureABC*), and an allophanate pathway which involves the ATP-dependent carboxylation to urea-1-carboxylate (allophanate) catalyzed by urea carboxylase, followed by hydrolysis to CO_2_ and ammonia by allophanate hydrolase (see Additional file 4: Figure S8). The latter pathway may be involved in the assimilation of urea as nitrogen source [88].

#### Taurine

*Arthrobacter* sp. strain Rue61a is unable to grow with taurine (2-aminoethanesulfonate) as sole carbon source or as sole source of nitrogen, but taurine supports growth when supplemented as sole sulfur source (Table 2). A putative taurine dioxygenase (TauD), which generates aminoacetaldehyde and sulfite, is encoded by ARUE_c31570; genes of a putative TauABC-like sulfonate transporter are located adjacently. Another possible *tauD* gene may be represented by ARUE_c20530. Genes of an alternative taurine degradation pathway via sulfo acetaldehyde and acetylphosphate were not identified in the genome.

## Conclusions

The genome of *Arthrobacter* sp. Rue61a contains a high number of genes coding for transporters presumably involved in the uptake of nutrients, and many genes coding for the utilization of carbohydrates, aromatic compounds deriving from lignin, and organic nitrogen compounds, consistent with the saprophytic lifestyle of the organism. Its nutritional versatility gives strain Rue61a a competitive advantage in soil environments. Genome analysis also suggests a strong adaptive potential to osmotic and oxidative stress, which presumably is the key to the environmental robustness of the genus. The genomes of strain Rue61a and of *A*. *aurescens* TC1 show a high overall similarity, and both strains are highly resistant to Pb^2+^ and arsenate. However, the two strains as well as the other environmental *Arthrobacter* strains whose genomes have been sequenced significantly differ in their biodegradative capabilities, presumably reflecting the adaptation of coexisting soil bacteria to distinct nutritional niches.

## Methods

### Bacterial strain

Strain Rue61a was isolated previously from sewage sludge for its ability to utilize quinaldine as carbon and energy source [25,26]. It was deposited into the DSMZ strain collection as strain DSM 24942. Based mainly on the 16S rRNA gene sequence, we had previously assigned it to the species *A*. *nitroguajacolicus*[17], however, its substrate utilization pattern differs from that of the type strain of *A*. *nitroguajacolicus* and also does not match the differentiating characteristics of the *A*. *aurescens* and *A*. *ilicis* type strains as reported by Kotoučková et al. [89], rendering the species allocation of strain Rue61a uncertain. The genome sequences of the type strains of *A*. *nitroguajacolicus* and *A*. *ilicis* are not available.

### Genome sequencing, assembly and gap closure

A combination of Sanger and pyrosequencing was used for whole-genome sequencing of *Arthrobacter* sp. Rue61a. The isolated DNA from strain Rue61a was used to create a 454-shotgun library following the GS Rapid library protocol (Roche 454, Branford, USA). The 454 DNA library was sequenced with the Genome Sequencer FLX (Roche 454) using Titanium chemistry. A total of 210092 shotgun reads were generated and assembled de novo into 92 large contigs (> 500 bp) using the Roche Newbler assembly software 2.3 (Roche 454). The final gap closure was done by PCR and primer walking using BioXact kit (Bioline GmbH, Germany) and the 5-prime Extender Polymerase System (5 PRIME GmbH, Hamburg, Germany) as described by the respective manufacturer. The Sanger sequencing approach was done using ABI 3730XL automated DNA sequencers (Life technologies, Darmstadt, Germany). Sequence editing was done by using GAP4 as part of Staden software package [90], processed with Phred and finally Phrap assembled ( http://www.phrap.org).

### Gene prediction and annotation and comparative genomics

Coding sequences (CDS) and open reading frames (ORFs) were predicted with YACOP [91] using the ORF finders Glimmer, Critica, and Z-curve. All CDS were manually curated and verified by using criteria such as the presence of a ribosome-binding site, GC frame plot analysis and comparison with the publicly available databases SwissProt, GenBank, ProDom, COG, and Prosite. The Genome annotation was performed automatically and annotation was manually curated. For the genome-wide identification of orthologs of two organisms, the BiBaG software tool was used (pers. comm. Antje Wollherr and Heiko Liesegang, Göttingen). BiBaG in a first step identifies bidirectional best BLAST hits. In a second step the proteins with bidirectional best BLAST hits are compared using the Needleman-Wunsch algorithm. For bidirectional BLAST searches, the BLAST default cut-off was used; all protein pairs with at least 30% sequence identity were subjected to further analysis.

### Pulsed field gel electrophoresis (PFGE)

Cell pellets of *Arthrobacter* sp. Rue61a grown in Lysogeny Broth (LB) were preincubated in PIV buffer (1 M NaCl, 1 mM Tris/HCl, pH 7.6) containing 1 mg lysozyme mL^–1^ (37°C, 15 min), embedded in low-melting-point agarose, lysed using the method of Schenk et al. [92], and equilibrated in TE buffer (10 mM Tris/HCl, 1 mM EDTA, pH 8). Subsequent proteinase K treatment of the agarose plugs was performed as described in [92], however, the samples were incubated for 5 h at 50°C instead of overnight. PFGE was carried out in a Rotaphor^TM^ PFGE system (Biometra) using broad range agarose gels (1%, w/v). Electrophoresis was performed at 6 V cm^–1^, 14°C, and a reorientation angle of 120°, in 0.3 × TBE (27 mM Tris/HCl, 27 mM boric acid, 0.6 mM EDTA). The switch time was increased linearly from 10 s to 40 s during the 24 h run. Concatemers of λ DNA were used as size standard (New England BioLabs Inc.).

### Metal susceptibility tests

The minimum inhibitory concentration (MIC) of metal ions and -oxides for *Arthrobacter* sp. strain Rue61a was determined by monitoring growth in half-concentrated LB (½LB: 2.5 g yeast extract, 5 g tryptone, 5 g NaCl per L) amended with metal salts (CoCl_2_: 0.016 – 20 mM, NiSO_4_: 0.016 – 20 mM, CuSO_4_: 0.016 – 20 mM, ZnSO_4_: 0.016 – 20 mM, CdSO_4_: 1.56 – 100 μM, K_2_CrO_4_: 0.016 – 20 mM, NaAsO_2_: 0.016 – 0.8 mM, Na_2_HAsO_4_: 0.08 – 1000 mM, HgCl_2_: 1.56 – 100 μM, and Pb(NO_3_)_2_: 0.06 – 5 mM). Cultures were inoculated with cell suspension from an exponential culture to an initial optical density at 600 nm (OD_600nm_) of 0.005–0.01, and growth was monitored by measuring turbidity with a Klett colorimeter (Manostat Corporation, New York, USA). For Pb(NO_3_)_2_, we also assessed its effect on the aerobic respiration of resting cells, because assays based on growth were reported to overestimate the resistance level due interactions of Pb^2+^ with media components [61]. *A*. *aurescens* TC1 and *Arthrobacter* sp. Rue61a were cultivated in LB with (10 μM) and without Pb(NO_3_)_2_, harvested by centrifugation in the late exponential phase, and resuspended in 10 mM 2-N-morpholinoethanesulfonic acid (MES) buffer (pH 6.5). The respiration rate of the cell suspension was measured with a Clark-type oxygen electrode (Rank Brothers LTD., Digital Model 10) at 25°C. The density of the cell suspension was adjusted to result in a linear decrease from 100%–10% O_2_ over 3–5 min (OD_600nm_: 2–5). Pb(NO_3_)_2_ was injected at an oxygen saturation of 80% and all measurements were repeated three times. The relative respiration rate was defined as ratio of the ΔO_2_ after and prior to Pb(NO_3_)_2_ injection. To the data from the experiments performed with suspensions of LB-grown cells, a dose–response equation was fit using Origin8G software: Relative respiration rate = k_1_ + (k_2_ – k_1_)/{1 + (10^(logIC50-[Pb(NO3)2])×p^); where k_1_ is the minimum respiration rate (at 1000 μM Pb(NO_3_)_2_) which defines 100% inhibition, k_2_ is the respiration rate without injection of Pb(NO_3_)_2_ (0% inhibition), which was set to 1, [Pb(NO3)2] is the concentration of Pb(NO_3_)_2_ in the assay, and p is the Hill-slope.

### Antibiotic susceptibility assays

The susceptibility of *Arthrobacter* sp. Rue61a to antibiotics was determined in agar diffusion tests. Cell suspensions (OD_600nm_: 0.8) were spread on Mueller-Hinton agar plates. After excess surface moisture had absorbed, filter discs (6 mm) soaked with test solution were placed on the plates. Antibiotics were tested in serial dilutions in the range of 0.1–100 μg/disk. Stock solutions of antibiotics were prepared in water, however, puromycin was dissolved in 0.5 M HEPES (pH 7.7). Plates were incubated at 30°C for 48 h and zones of inhibition were measured from the edge of the paper disk to the area of visible growth. All assays were performed in triplicate.

### Substrate utilization assays

Growth of strain Rue61a on aromatic and *N*-heterocyclic compounds, carboxylic acids, alcohols, sugars (and derivatives), 1-oleyl-*rac*-glycerol, nitrogen compounds and the aminoethanesulfonate taurine was tested in liquid cultures in mineral salts medium (MM) [17]. For substrates with poor water solubility, stock solutions were prepared in DMSO. 1 *H*-3-Hydroxy-4-oxoquinaldine was synthesized from 1 *H*-4-oxoquinaldine via 1 *H*-3-formyl-4-oxoquinaldine according to [93,94], and HHQ and PQS were produced by biotransformation as described in [95]. Media were prepared in baffled Erlenmeyer flasks, inoculated from glucose-grown pre-cultures to an initial OD_600nm_ of 0.05–0.1, and incubated with shaking at 30°C. After reaching the stationary phase, aliquots were transferred into fresh medium containing the respective substrate; if appropriate, repeated passages were performed to confirm growth. For all growth tests, uninoculated controls as well as cultures lacking the substrate to be tested were run in parallel. Growth was monitored by measuring OD_600nm_ at appropriate time intervals. For substrates that exhibit a distinct light absorption spectrum, substrate consumption and formation of any metabolites was followed by recording UV/Vis-spectra of culture supernatants at appropriate time intervals. For testing the utilization of substrates as sole nitrogen or sulfur sources, glucose (0.5%) was used as carbon source.

To find out whether growth in the presence of individual aromatic compounds affects the activity of catechol oxidizing enzyme(s) of strain Rue61a, cultures were pre-grown in MM containing glucose (0.5%) and 1 mM or 2 mM of the aromatic substrate, harvested by centrifugation, and re-suspended to an OD_600nm_ of ~20 in MM containing 1 mM catechol. At appropriate time intervals, samples were collected, centrifuged, and the UV spectra of the supernatants were recorded.

The utilization and hydrolysis of polysaccharides, protein and lipid was assessed in agar plate assays. Starch (0.3% and 1%), lichenin (0.02%), carboxymethyl cellulose (0.01% and 1%), chitin (0.1%), pectin (0.1%), skim milk powder (2%), and tributyrin (1%) were suspended in MM or ½LB agar, and the plates were inoculated with 10 μl aliquots of cell suspension from an overnight culture. Cultures on tributyrin, lichenin, chitin and skim milk plates were checked for growth (MM) and zones of clearing around the colonies (MM and ½LB). Cultures on plates containing starch, carboxymethylcellulose and pectin were covered with Lugol's solution (6.8 g L^–1^ KI, 3.4 g L^–1^ I_2_), Congo red (0.1%, w/v), and copper acetate (10%, w/v), respectively.

### Expression of genes coding for anthranilate-CoA ligases, and activity assay

For analysis of the putative anthranilate-CoA ligases encoded by ARUE_113p00220 and ARUE_c41730, the genes were PCR amplified using the primer pairs ATATCATATGACCTCCACATCATCGGCC/ATATCTCGAGTGGCTGGGCGGACGCGCCGG and ATATCATATGAGCATGTTGCCATCGG/ATATCTCGAGGGCCTGGCTCTGGCCGGCG, respectively, using genomic DNA as template. The purified PCR products were digested with *Nde*I and *Xho*I and ligated into pET22b(+), and competent cells of *E*. *coli* Rosetta(DE3)pLysS (Novagen) were transformed with the ligation mixtures. The plasmid inserts were verified by sequencing (GATC Biotech AG, Konstanz, Germany). The recombinant strains were grown in LB with ampicillin (100 μg mL^–1^) and chloramphenicol (34 μg mL^–1^) at 37°C. At an OD_600nm_ of 0.5, isopropyl-*β*-D-thiogalactopyranosid (0.5 mM) was added, the cells were grown for another 6 h at 30°C, and harvested by centrifugation. Cells suspended in buffer (100 mM Tris/HCl pH 7.8, 2 mM MgCl_2_), supplemented with 12.5 U mL^–1^ Benzonase (Novagen), were disrupted by sonication, and cell extract supernatant was obtained by centrifugation. Anthranilate-CoA ligase activity in the supernatants was determined spectrophotometrically by measuring formation of anthraniloyl-CoA at 365 nm (ε_365 nm_ = 5,500 M^–1^ cm^–1^; [96]) at 30°C. The assay contained 100 mM Tris/HCl (pH 7.8), 0.2 mM dithiothreitol, 2 mM MgCl_2_, 1 mM ATP, 0.4 mM coenzyme A, and 1 mM anthranilic acid. One Unit was defined as the amount of enzyme that catalyzes the formation of 1 μmol of anthraniloyl-CoA per minute. The identity of the product was confirmed by mass spectrometry. Protein concentrations in cell extract supernatants were estimated using a modified Bradford assay [97].

### LC-MS analysis of CoA thioesters in cell extracts of *Arthrobacte*r sp. Rue61a

*Arthrobacter* sp. strain Rue61a was cultivated in mineral salts medium [17] supplemented with 1 *H*-4-oxoquinaldine (2 mM), anthranilate (2 mM), benzoate (2 mM) plus glucose (0.5%), or glucose (4%). Culture samples were harvested, concentrated to an OD_600 nm_ of 30, and the suspension (1 mL) was quenched with 4.5 mL of 25 mM formic acid in acetonitrile at –20°C. The samples were incubated for 15 min on ice with occasional mixing, chilled with liquid nitrogen, and lyophilized. Samples were dissolved in 150 μL ammonium formate buffer (50 mM, pH 3.5, 2% methanol). After mixing, samples were centrifuged twice (20,000 × *g*, 5 min, 4°C) and the supernatant was used for analysis. LC-MS analyses were performed with a Rheos 2200 HPLC system (Flux Instruments, Basel, Switzerland) coupled to an LTQ Orbitrap mass spectrometer (Thermo Fisher Scientific, Waltham, MA, USA), equipped with an electrospray ionization probe. Coenzyme A esters were analyzed as described previously [98] with slight modifications [99]. First, potential CoA esters were identified applying a general approach as described previously [99]. In a second identification approach, samples were spiked with uniformly ^13^C labeled cell extract from *Methylobacterium extorquens* AM1 as internal standard. CoA esters of *M*. *extorquens* have been extensively characterized previously [98].

### Nucleotide sequence accession number

The complete genome sequence of *Arthrobacter* sp. Rue61a was deposited in GenBank under the accession numbers [GenBank:CP003203]–[Genbank:CP003205].

## Competing interests

The authors declare that they have no competing interests.

## Authors’ contributions

HN designed and performed physiological experiments. JS performed sequencing and bioinformatic analysis of sequence data. KP designed and performed cloning and PFGE experiments. PK and JAV designed and performed LC-MS experiments. JS and SF contributed to manual curation of the annotation of the genome sequence. RD supervised sequencing work and analysis. SF supervised experimental work. SF wrote the draft of the manuscript, all other authors contributed to subsequent revisions to the final version. All authors read and approved the final version of the manuscript.

## Supplementary Material

Additional file 1 **Table S1.** Genes of Arthrobactersp. Rue61as associated with putative genomic islands. Click here for file

Additional file 2 **Tables S2.** Putative transporters and binding proteins of Arthrobactersp. Rue61a. Click here for file

Additional file 3 **Tables S3.** Genes of *Arthrobacter* sp. Rue61a, grouped according to functions, as discussed in the text. Click here for file

Additional file 4 **Figures S1.** Biodegradation pathways of *Arthrobacter* sp. Rue61a. **Figures S2.** Degradation of 4-hydroxybenzoate, vanillate, and protocatechuate via the *ortho* pathway. **Figures S3.** Degradation of 4-hydroxyphenylacetate and homoprotocatechuate via the *meta* pathway. **Figures S4.** Choline, creatine and sarcosine metabolism. **Figures S5.** Agmatine and putrescine degradation. **Figures S6.** Oxidation of hypoxanthine and xanthine to urate and degradation to allantoin. **Figures S7.** Allantoin degradation to glyoxylate, glyoxylate metabolism via the D-glycerate pathway. **Figures S8.** Urea degradation. Click here for file

Additional file 5 **Table S4.** LC-MS analysis of Coenzyme A thioesters in cell extracts of *Arthrobacter* sp. Rue61a. Click here for file
