# Supplementary material for: Complete genome sequence and metabolic potential of the quinaldine-degrading bacterium Arthrobacter sp. Rue61a
Source: BMC Genomics. 2012 Oct 6;13:534. doi: 10.1186/1471-2164-13-534 (PMC3534580; doi:10.1186/1471-2164-13-534)
Supplement: Additional file 1 — Table S1. Genes of Arthrobactersp. Rue61as associated with putative genomic islands. [file 1471-2164-13-534-S1.pdf]

Additional File 1:

Genes of *Arthrobacter* sp. Rue61a associated with putative genomic islands

Table S1: Genes of *Arthrobacter* sp. Rue61a associated with putative genomic islands, generated by IslandViewer web-tool.

**Locus_tag**	**Annotation**
ARUE_c17500	hypothetical protein
ARUE_c17510	hypothetical protein
ARUE_c17520	nephrocystin
ARUE_c17530	hypothetical protein
ARUE_c17540	hypothetical protein
ARUE_c17550	hypothetical protein
ARUE_c17560	hypothetical protein
ARUE_c17570	hypothetical protein
ARUE_c17580	hypothetical protein
ARUE_c17590	hypothetical protein
ARUE_c17600	hypothetical protein
ARUE_c17610	hypothetical protein
ARUE_c17620	hypothetical protein
ARUE_c17630	hypothetical protein
ARUE_c17640	hypothetical protein
ARUE_c17650	possible sugar phosphate isomerase/ epimerase
ARUE_c18300	ferredoxin-2
ARUE_c18310	putative cytochrome P450 (CYP) oxygenase
ARUE_c18320	putative ferredoxin reductase / NAD(FAD)-dependent dehydrogenase
ARUE_c18330	EmrB/QacA subfamily drug resistance transporter
ARUE_c18340	regulatory protein, TetR
ARUE_c18350	2-(S)-hydroxypropyl-CoM dehydrogenase XecE
ARUE_c18360	medium-chain dehydrogenase/reductase (MDR) family protein
ARUE_c18370	fumarylacetoacetate (FAA) superfamily protein
ARUE_c18410	D-lactate dehydrogenase
ARUE_c18420	dihydroxy-acid dehydratase IlvD
ARUE_c18430	hypothetical protein
ARUE_c18440	extracellular beta-glucosidase BglX
ARUE_c18450	putative rhamnosidase
ARUE_c18460	hypothetical protein
ARUE_c18470	glucose-resistance amylase regulator
ARUE_c18480	putative sugar ABC transporter, permease protein
ARUE_c18490	putative sugar ABC transporter, permease protein
ARUE_c18500	putative secreted sugar-binding protein
ARUE_c18510	ABC transporter
ARUE_c18520	alpha-L-rhamnosidase
ARUE_c18550	hypothetical protein
ARUE_c18560	putative NAD(P)-dependent oxidoreductase, short chain dehydrogenase/reductase family
ARUE_c18570	putative glucitol transport protein GutA
ARUE_c18580	transcriptional regulatory protein
ARUE_c18590	hypothetical protein

ARUE_c18600	6-phosphogluconate dehydrogenase NAD-binding
ARUE_c18610	hypothetical protein
ARUE_c18620	triosephosphate isomerase
ARUE_c18630	putative sugar phosphate isomerase
ARUE_c18640	putative dihydroxyacetone kinase
ARUE_c18650	putative tartrate transporter TtuB
ARUE_c18660	GntR domain protein
ARUE_c18670	transcriptional regulator, TetR family
ARUE_c18680	major facilitator superfamily MFS_1
ARUE_c18690	3-alpha-(or 20-beta)-hydroxysteroid dehydrogenase FabG
ARUE_c18700	thermostable beta-glucosidase B
ARUE_c18710	putative inosine-uridine preferring nucleoside hydrolase
ARUE_c18720	extracellular beta-glucosidase BglX
ARUE_c18730	hypothetical protein
ARUE_c18740	hypothetical protein
ARUE_c18750	hypothetical protein
ARUE_c18760	putative oxidoreductase
ARUE_c18770	transcriptional regulator, TetR family
ARUE_c18780	3-hydroxybutyryl-CoA dehydratase Crt
ARUE_c18790	hypothetical protein
ARUE_c18800	putative transcriptional regulator
ARUE_c18810	uncharacterized protein YraM
ARUE_c18820	aldehyde dehydrogenase
ARUE_c18830	formyl-coenzyme A transferase Frc
ARUE_c18840	hypothetical protein
ARUE_c18850	hypothetical protein
ARUE_c18860	hypothetical protein
ARUE_c18870	putative cytochrome P450
ARUE_c18880	hypothetical protein
ARUE_c18890	hypothetical protein
ARUE_c18900	uncharacterized NAD(FAD) dependent dehydrogenase, ferredoxin reductase-like
ARUE_c18910	hypothetical protein
ARUE_c18920	hypothetical protein
ARUE_c18930	hypothetical protein
ARUE_c18940	hypothetical protein
ARUE_c18950	hypothetical protein
ARUE_c18960	hypothetical protein
ARUE_c18970	putative nucleic-acid-binding protein containinga Zn-ribbon
ARUE_c18980	putative 3-oxoacyl-[acyl-carrier-protein] reductase
ARUE_c18990	hypothetical protein
ARUE_c19000	hypothetical protein
ARUE_c19010	hypothetical protein
ARUE_c19020	putative enoyl-CoA hydratase/isomerase
ARUE_c19030	hypothetical protein
ARUE_c19040	hypothetical protein
ARUE_c19080	proline/betaine transporter ProP
ARUE_c19090	putative PucR-like transcriptional regulator
ARUE_c19100	hypothetical protein
ARUE_c19110	hypothetical protein
ARUE_c19120	hypothetical protein
ARUE_c19130	integral membrane protein
ARUE_c19140	putative integral membrane protein
ARUE_c19150	aminobenzoyl-glutamate utilization proteinB

ARUE_c19160	hypothetical protein
ARUE_c19240	transcriptional regulator, IclR family
ARUE_c19250	putative ABC transporter permease protein YesQ
ARUE_c19260	sn-glycerol-3-phosphate transport system permease protein UgpA
ARUE_c19270	3',5'-cyclic-nucleotide phosphodiesterase
ARUE_c19280	hypothetical protein
ARUE_c19290	putative ABC transporter substrate-binding protein
ARUE_c19300	sn-glycerol-3-phosphate import ATP-bindingprotein UgpC
ARUE_c19310	putative RpiR family transcriptional regulator
ARUE_c19320	hypothetical protein, hotdog fold superfamily
ARUE_c19330	hypothetical protein
ARUE_c19340	hypothetical protein
ARUE_c19350	hypothetical protein
ARUE_c19360	hypothetical protein
ARUE_c21000	HTH-type transcriptional regulator CynR
ARUE_c21020	HTH-type transcriptional regulator GbpR
ARUE_c21030	putative acid-CoA ligase
ARUE_c21040	hypothetical protein
ARUE_c21050	shikimate transporter ShiA
ARUE_c21060	cyclase family protein
ARUE_c21070	thiamine pyrophosphate-requiring enzyme
ARUE_c21080	amylo-alpha-1,6-glucosidase
ARUE_c21110	putative succinate-semialdehyde dehydrogenase [NAD(P)+]
ARUE_c21120	putative 4-aminobutyrate transaminase
ARUE_c21130	putative monomeric sarcosine oxidase SoxA
ARUE_c21140	glutamine transport ATP-binding protein GlnQ
ARUE_c21150	inner membrane amino-acid ABC transporter permease protein YecS
ARUE_c21160	ABC-type amino acid transport/signal transduction system, extracellular
ARUE_c21170	putative L-2-haloacid dehalogenase, type II
ARUE_c21180	FAD-dependent oxidoreductase
ARUE_c21190	NAD(P)+-dependent aldehyde-dehydrogenase superfamily protein
ARUE_c33020	putative uncharacterized DUF218 domain protein
ARUE_c33030	hypothetical protein
ARUE_c33040	O-antigen polymerase
ARUE_c33050	putative tyrosine-protein kinase YveL
ARUE_c33060	hypothetical protein
ARUE_c33070	putative NAD(P)H:flavin oxidoreductase / NAD(P)H nitroreductase
ARUE_c33080	hypothetical protein
ARUE_c33090	possible serine O-acetyltransferase
ARUE_c33100	putative glycosyltransferase
ARUE_c33110	glycosyl transferase, WecG/TagA family
ARUE_c33120	GDP-mannose 4,6 dehydratase
ARUE_c33130	GDP-L-fucose synthase
ARUE_c33140	putative undecaprenylphosphate glycosylphosphotransferase
ARUE_c33150	UDP-glucose 6-dehydrogenase RkpK
ARUE_c33160	hypothetical protein
ARUE_c36190	hypothetical protein
ARUE_c36200	hypothetical protein
ARUE_c36220	hypothetical protein
ARUE_c36230	hypothetical protein
ARUE_c36240	hypothetical protein
ARUE_c36250	hypothetical protein
ARUE_c36260	hypothetical protein

ARUE_c36270	hypothetical protein
ARUE_c36280	hypothetical protein
ARUE_c37290	transcriptional regulator, AraC family domain
ARUE_c37300	hypothetical membrane protein
ARUE_c37310	putative xylose repressor XylR
ARUE_c37320	D-3-phosphoglycerate dehydrogenase SerA
ARUE_c37330	N-sulphoglucosamine sulphohydrolase SGSH
ARUE_c37340	ABC-type sugar transport system extracellular component
ARUE_c37350	ABC transporter permease protein
ARUE_c37360	L-arabinose transport system permease protein AraQ
ARUE_c37370	putative dehydrogenase
ARUE_c37380	glucosamine-6-phosphate deaminase NagB
