# Supplementary material for: Complete genome sequence and metabolic potential of the quinaldine-degrading bacterium Arthrobacter sp. Rue61a
Source: BMC Genomics. 2012 Oct 6;13:534. doi: 10.1186/1471-2164-13-534 (PMC3534580; doi:10.1186/1471-2164-13-534)
Supplement: Additional file 2 — Tables S2. Putative transporters and binding proteins of Arthrobactersp. Rue61a. [file 1471-2164-13-534-S2.pdf]

Additional File 2:

Transporters and binding proteins of *Arthrobacter* sp. strain Rue61a

Table S2.1: Putative transporters and binding proteins of *Arthrobacter* sp. Rue61a with orthologs in *A. aurescens* TC1.

Locus_tag Rue61a	Annotation	Evalue	Locus_tag TC1
ARUE_c00100	integral membrane protein DUF6 domain protein	8.00E-167	AAur_0012
ARUE_c00420	putative amino acid permease	0	AAur_0045
ARUE_c00490	YhjE1:inner membrane metabolite transport protein YhjE	0	AAur_0052
ARUE_c00540	YocS:uncharacterized sodium-dependent transporter YocS	2.00E-178	AAur_0057
ARUE_c00550	YbaR:putative sulfate transporter YbaR	0	AAur_0058
ARUE_c00740	putative major facilitator superfamily (MFS) transporter	0	AAur_0078
ARUE_c00840	AraQ1:L-arabinose transport system permease protein AraQ	1.00E-170	AAur_0086
ARUE_c00850	LacF1:lactose transport system permease protein LacF	0	AAur_0087
ARUE_c00860	putative extracellular sugar-binding protein	0	AAur_0088
ARUE_c00880	RutG:putative pyrimidine permease RutG	0	AAur_0090
ARUE_c00950	YclF:uncharacterized transporter YclF	0	AAur_0097
ARUE_c01450	EmrB/QacA subfamily drug resistance transporter	0	AAur_0148
ARUE_c01550	putative integral membrane protein DUF6	6.00E-149	AAur_0160
ARUE_c01840	putative amino acid permease	0	AAur_0190
ARUE_c01890	PstS:phosphate-binding protein PstS	0	AAur_0195
ARUE_c01900	PstC:phosphate transport system permease protein PstC	1.00E-156	AAur_0196
ARUE_c01910	PstA:phosphate transport system permease protein PstA	0	AAur_0197
ARUE_c01920	PstB:phosphate import ATP-binding protein PstB (3.6.3.27)	2.00E-147	AAur_0198
ARUE_c01930	Pit1:putative low-affinity inorganic phosphate transporter Pit	0	AAur_0199
ARUE_c02150	putative high-affinity nickel-transport protein	0	AAur_0222
ARUE_c02350	YesQ1:putative ABC transporter permease protein YesQ	3.00E-174	AAur_0238
ARUE_c02360	YesP1:putative ABC transporter permease protein YesP	0	AAur_0239
ARUE_c02540	YgbN1:inner membrane permease YgbN	0	AAur_0256
ARUE_c02760	YdeK:uncharacterized transporter YdeK	8.00E-170	AAur_0278
ARUE_c02780	ProP1:proline/betaine transporter ProP	0	AAur_0280

ARUE_c02910	HPr2:phosphocarrier protein HPr	4.00E-45	AAur_0293
ARUE_c02920	FruA:PTS system fructose-specific EIIABC component (2.7.1.69)	0	AAur_0294
ARUE_c03060	EmrB/QacA subfamily drug resistance transporter	4.00E-79	AAur_pTC20115
ARUE_c03350	putative transmembrane efflux protein	0	AAur_0362
ARUE_c03440	AzlC:putative azaleucine resistance protein AzlC	6.00E-145	AAur_0371
ARUE_c03540	MsbA1:lipid A export ATP-binding/permease protein MsbA	0	AAur_0381
ARUE_c03550	putative ABC transporter, ATP-binding protein	0	AAur_0383
ARUE_c03590	Fe3+-siderophore ABC transporter, transmembrane subunit	0	AAur_0387
ARUE_c03600	Fe3+-siderophore ABC transporter, transmembrane subunit	0	AAur_0388
ARUE_c03610	Fe3+-siderophore ABC transporter, ATP-binding protein	2.00E-155	AAur_0389
ARUE_c03630	ShiA1:shikimate transporter ShiA	0	AAur_0391
ARUE_c03660	ProY:proline-specific permease ProY	0	AAur_0394
ARUE_c03790	putative permease	0	AAur_0406
ARUE_c03850	CzcD:putative cation (cobalt-zinc-cadmium) efflux transporter CzcD	2.00E-170	AAur_0412
ARUE_c03880	YdfC:uncharacterized transporter YdfC	6.00E-177	AAur_0415
ARUE_c04120	YurO1:uncharacterized ABC transporter extracellular- binding protein YurO	0	AAur_0443
ARUE_c04130	YurN1:putative ABC transporter permease protein YurN	0	AAur_0444
ARUE_c04140	YurM1:putative ABC transporter permease protein YurM	5.00E-157	AAur_0445
ARUE_c04280	AglE:alpha-glucosides-binding extracellular protein AglE	0	AAur_0459
ARUE_c04290	AglF:alpha-glucoside transport system permease protein AglF	5.00E-179	AAur_0460
ARUE_c04300	AglG:alpha-glucoside transport system permease protein AglG	0	AAur_0461
ARUE_c04460	betaine/carnitine/choline transporter (BCCT) family protein	0	AAur_0477
ARUE_c04820	putative amino acid permease	0	AAur_0511
ARUE_c04920	putative extracellular sugar-binding protein	0	AAur_0521
ARUE_c04930	putative ABC transporter permease protein y4oQ	0	AAur_0522
ARUE_c04940	YcjP:inner membrane ABC transporter permease protein YcjP	1.00E-153	AAur_0523
ARUE_c05020	ChvE:multiple sugar-binding extracellular receptor ChvE	0	AAur_0531
ARUE_c05030	AraG1:L-arabinose transport ATP-binding protein AraG (3.6.3.17)	0	AAur_0532
ARUE_c05040	XylH1:xylose transport system permease protein XylH	0	AAur_0533
ARUE_c05080	YhaQ:uncharacterized ABC transporter ATP-binding protein YhaQ	0	AAur_0537
ARUE_c05090	putative membrane protein	9.00E-166	AAur_0538
ARUE_c05260	Fe3+-siderophore ABC transporter, ATP-binding protein	2.00E-152	AAur_0557
ARUE_c05270	Fe3+-siderophoreABC transporter, transmembrane subunit	0	AAur_0558

ARUE_c05280	Fe3+-siderophore ABC transporter, transmembrane subunit	0	AAur_0559
ARUE_c05290	Fe3+-siderophore binding protein	0	AAur_0560
ARUE_c05340	EmrB/QacA subfamily drug resistance transporter	0	AAur_0565
ARUE_c05460	ProP2:proline/betaine transporter ProP	0	AAur_0577
ARUE_c05510	putative amino acid permease	0	AAur_0582
ARUE_c05520	ShiA2:shikimate transporter ShiA	0	AAur_0583
ARUE_c05550	putative sodium/hydrogen exchanger family protein	0	AAur_0586
ARUE_c05620	ArgT:lysine-arginine-ornithine-binding extracellular protein ArgT	1.00E-155	AAur_0593
ARUE_c05630	ArtQ1:arginine transport system permease proteinArtQ	7.00E-144	AAur_0594
ARUE_c05640	GlnQ1:glutamine transport ATP-binding protein GlnQ	6.00E-146	AAur_0595
ARUE_c05800	putative extracellular solute-binding domain protein	0	AAur_0612
ARUE_c05810	YurN2:putative ABC transporter permease protein YurN	0	AAur_0613
ARUE_c05820	YurM2:putative ABC transporter permease protein YurM	3.00E-164	AAur_0614
ARUE_c05890	YhjE2:inner membrane metabolite transport protein YhjE	0	AAur_0621
ARUE_c06090	YehY:putative osmoprotectant uptake system permease protein YehY	9.00E-128	AAur_0644
ARUE_c06100	OpuCA1:glycine betaine/carnitine/choline transport ATP- binding protein OpuCA	0	AAur_0645
ARUE_c06110	OpuBB:choline transport system permease protein OpuBB	1.00E-142	AAur_0646
ARUE_c06120	OsmF:putative osmoprotectant uptake system substrate- binding protein OsmF	3.00E-179	AAur_0647
ARUE_c06230	putative major facilitator superfamily (MFS) transporter	0	AAur_0659
ARUE_c06370	EmrB/QacA subfamily drug resistance transporter	0	AAur_0673
ARUE_c06490	extracellular solute-binding domain protein	0	AAur_0685
ARUE_c06500	YurN3:putative ABC transporter permease protein YurN	2.00E-177	AAur_0686
ARUE_c06510	AraQ2:L-arabinose transport system permease protein AraQ	8.00E-169	AAur_0687
ARUE_c06610	putative ABC transporter permease protein y4oR	3.00E-160	AAur_0697
ARUE_c06620	putative ABC transporter permease protein y4oQ	4.00E-167	AAur_0698
ARUE_c06630	putative extracellular sugar-binding protein	0	AAur_0699
ARUE_c06660	uncharacterized 52.8 kDa protein in TAR-I ttuC' 3'region	0	AAur_0702
ARUE_c06670	putative integral membrane protein	2.00E-91	AAur_0703
ARUE_c06680	YflP:UPF0065 protein YflP	0	AAur_0704
ARUE_c06820	putative major facilitator superfamily (MFS) transporter	0	AAur_0720
ARUE_c06900	putative TRAP transporter solute receptor, TAXI family	1.00E-180	AAur_0728

ARUE_c06930	KgtP1:alpha-ketoglutarate permease KgtP	0	AAur_0731
ARUE_c07040	putative Fe(3+) transport system, ATP binding protein (3.6.3.30)	0	AAur_0751
ARUE_c07050	putative Fe(3+)-transport system, permease protein (3.6.3.30)	0	AAur_0752
ARUE_c07060	putative Fe(3+)-binding extracellular protein (3.6.3.30)	0	AAur_0753
ARUE_c07590	EamA:putative amino-acid metabolite efflux pump	7.00E-169	AAur_0808
ARUE_c07630	GsiB1:glutathione-binding protein GsiB	0	AAur_0812
ARUE_c07640	GsiC:glutathione transport system permease protein GsiC	0	AAur_0813
ARUE_c07650	GsiD1:glutathione transport system permease protein GsiD	8.00E-160	AAur_0814
ARUE_c07660	GsiA1:glutathione import ATP-binding protein GsiA	0	AAur_0815
ARUE_c07710	hypothetical protein	6.00E-149	AAur_0820
ARUE_c07770	AppF1:oligopeptide transport ATP-binding proteinAppF	3.00E-150	AAur_0826
ARUE_c07780	putative peptide import ATP-binding protein	0	AAur_0827
ARUE_c07790	putative peptide transport system permease protein	5.00E-166	AAur_0828
ARUE_c07800	GsiB2:glutathione-binding protein GsiB	0	AAur_0829
ARUE_c07890	HsrA:putative transport protein HsrA	0	AAur_0838
ARUE_c07930	CycA1:D-serine/D-alanine/glycine transporter CycA	0	AAur_0842
ARUE_c08260	RbsB1:D-ribose-binding extracellular protein RbsB	3.00E-174	AAur_0873
ARUE_c08270	RbsA1:ribose import ATP-binding protein RbsA (3.6.3.17)	0	AAur_0874
ARUE_c08280	RbsC1:ribose transport system permease protein RbsC	6.00E-174	AAur_0875
ARUE_c08320	YdhP1:inner membrane transport protein YdhP	0	AAur_0879
ARUE_c08710	MalE:maltose-binding protein MalE	0	AAur_0918
ARUE_c08720	MalF:maltose transport system permease protein MalF	0	AAur_0919
ARUE_c08730	GanQ:putative arabinogalactan oligomer transport system permease protein GanQ	1.00E-172	AAur_0920
ARUE_c08760	putative major facilitator superfamily (MFS) transporter	0	AAur_0923
ARUE_c08820	UgpC1:sn-glycerol-3-phosphate import ATP- bindingprotein UgpC (3.6.3.20)	0	AAur_0929
ARUE_c08950	YurM3:putative ABC transporter permease protein YurM	2.00E-167	AAur_0944
ARUE_c08960	YurN4:putative ABC transporter permease protein YurN	6.00E-166	AAur_0945
ARUE_c08970	putative extracellular sugar-binding protein	0	AAur_0946
ARUE_c09170	ShiA3:shikimate transporter ShiA	0	AAur_0965
ARUE_c09250	putative amino acid permease	0	AAur_0973
ARUE_c09360	EmrB/QacA subfamily drug resistance transporter	0	AAur_0985

ARUE_c09460	putative membrane protein	0	AAur_0995
ARUE_c09570	putative amino-acid transporter	1.00E-114	AAur_1006
ARUE_c09670	putative transmembrane efflux protein (MFS)	0	AAur_1015
ARUE_c09820	TcyC1:L-cystine import ATP-binding protein TcyC	4.00E-149	AAur_1028
ARUE_c09830	TcyB:L-cystine transport system permease protein TcyB	9.00E-121	AAur_1029
ARUE_c09840	TcyA:L-cystine-binding protein TcyA	4.00E-149	AAur_1030
ARUE_c09870	ABC transporter F family member	0	AAur_1033
ARUE_c09880	YgaD1:putative multidrug export ATP-binding/permease protein YgaD	0	AAur_1034
ARUE_c10090	putative sodium:solute symporter family protein	0	AAur_1054
ARUE_c10160	MFS-type transporter	0	AAur_1061
ARUE_c10170	ABC transporter ATP-binding protein	0	AAur_1062
ARUE_c10180	ABC transporter ATP-binding protein	0	AAur_1063
ARUE_c10360	KgtP2:alpha-ketoglutarate permease KgtP	0	AAur_1081
ARUE_c10450	putative membrane protein	0	AAur_1092
ARUE_c10590	YfaV:inner membrane transport protein YfaV	0	AAur_1107
ARUE_c10760	putative major facilitator superfamily (MFS) transporter	0	AAur_1124
ARUE_c10800	LplB1:protein LplB	0	AAur_1128
ARUE_c10810	LplC:protein LplC	7.00E-159	AAur_1129
ARUE_c10820	extracellular solute-binding domain protein	0	AAur_1130
ARUE_c10970	YwoD:uncharacterized MFS-type transporter YwoD	0	AAur_1146
ARUE_c11050	PuuP:putrescine importer PuuP	0	AAur_1167
ARUE_c11140	uncharacterized transporter Mb2022c	0	AAur_1180
ARUE_c11150	MFS-type transporter	0	AAur_1179
ARUE_c11280	YceI1:putative metabolite transport protein YceI	0	AAur_1194
ARUE_c11390	PhnE:putative phosphonates transport system permease protein PhnE	4.00E-157	AAur_1203
ARUE_c11400	PhnC:phosphonates import ATP-binding protein PhnC (3.6.3.28)	3.00E-144	AAur_1204
ARUE_c11410	PhnD:phosphate-binding protein of phosphonate ABC transporter PhnD	1.00E-169	AAur_1205
ARUE_c11470	ABC transporter ATP-binding protein	6.00E-126	AAur_1211
ARUE_c11480	putative ABC transporter permease protein	0	AAur_1212
ARUE_c11490	sugar-binding extracellular receptor protein of ABC transporter system	0	AAur_1213
ARUE_c11500	XylH2:xylose transport system permease protein XylH	0	AAur_1214
ARUE_c11510	AraG2:L-arabinose transport ATP-binding protein AraG (3.6.3.17)	0	AAur_1215
ARUE_c11560	YhdG1:uncharacterized amino acid permease YhdG	0	AAur_1220

ARUE_c11650	NodI1:Nod factor export ATP-binding protein I	9.00E-174	AAur_1229
ARUE_c11660	putative ABC-2 type transporter	2.00E-151	AAur_1230
ARUE_c11670	NodJ:nodulation protein J	1.00E-157	AAur_1231
ARUE_c11680	ShiA4:shikimate transporter ShiA	0	AAur_1232
ARUE_c11780	YesO2:putative ABC transporter substrate-binding protein YesO	0	AAur_1242
ARUE_c11890	putative basic membrane lipoprotein	0	AAur_1253
ARUE_c11900	YufO:uncharacterized ABC transporter ATP-binding protein YufO	0	AAur_1254
ARUE_c11910	YufP:uncharacterized ABC transporter permease protein YufP	0	AAur_1255
ARUE_c11920	YufQ:uncharacterized ABC transporter permease protein YufQ	4.00E-177	AAur_1256
ARUE_c12070	BraC:branched-chain amino acid ABC transporter BraC	0	AAur_1271
ARUE_c12090	BraG:high-affinity branched-chain amino acid transport ATP-binding protein BraG	5.00E-139	AAur_1273
ARUE_c12100	LivG:putative branched-chain amino acid transport ATP- binding protein LivG	0	AAur_1274
ARUE_c12110	BraE:high-affinity branched-chain amino acid transport system permease protein BraE	0	AAur_1275
ARUE_c12120	inner-membrane translocator	4.00E-165	AAur_1276
ARUE_c12340	MFS-type transporter	0	AAur_1297
ARUE_c12760	YfmR:uncharacterized ABC transporter ATP-binding protein YfmR	0	AAur_1339
ARUE_c12900	NhaA:Na(+)/H(+) antiporter NhaA	0	AAur_1352
ARUE_c13010	putative cation efflux family protein	2.00E-134	AAur_1364
ARUE_c13060	YfiG:putative metabolite transport protein YfiG	0	AAur_1368
ARUE_c13240	nitrite extrusion protein	0	AAur_1408
ARUE_c13250	YhjE3:inner membrane metabolite transport protein YhjE	0	AAur_1409
ARUE_c13280	YdfJ:membrane protein YdfJ	0	AAur_1412
ARUE_c13310	YdjN:uncharacterized symporter YdjN	0	AAur_1415
ARUE_c13540	SsuB:aliphatic sulfonates import ATP-binding protein SsuB	4.00E-134	AAur_1439
ARUE_c13550	SsuC1:putative aliphatic sulfonates transport permease protein SsuC	7.00E-173	AAur_1440
ARUE_c13560	putative ABC-type sulfonate transport system	0	AAur_1441
ARUE_c13610	CitN:citrate transporter CitN	0	AAur_1446
ARUE_c13700	LplB2:protein LplB	0	AAur_1453
ARUE_c13710	YtcP:putative ABC transporter permease protein YtcP	3.00E-173	AAur_1454
ARUE_c14090	YdgK:uncharacterized MFS-type transporter YdgK	0	AAur_1495

ARUE_c14240	putative sodium:solute symporter family protein	0	AAur_1510
ARUE_c14290	YwcA:uncharacterized symporter YwcA	0	AAur_1515
ARUE_c14510	putative ABC transporter permease protein	2.00E-171	AAur_1538
ARUE_c14520	YurN5:putative ABC transporter permease protein YurN	1.00E-174	AAur_1539
ARUE_c14530	YurO2:uncharacterized ABC transporter extracellular- binding protein YurO	0	AAur_1540
ARUE_c14630	putative ABC transporter efflux protein, DrrB family	3.00E-133	AAur_1550
ARUE_c14640	NodI2:Nod factor export ATP-binding protein I	2.00E-138	AAur_1551
ARUE_c15210	putative major facilitator superfamily (MFS) transporter	0	AAur_1610
ARUE_c15270	SLC30A9: zinc transporter 9	8.00E-169	AAur_1616
ARUE_c15280	YdhP2:inner membrane transport protein YdhP	0	AAur_1617
ARUE_c15780	DctA1:C4-dicarboxylate transport protein	0	AAur_1668
ARUE_c15790	hypothetical protein	2.00E-166	AAur_1669
ARUE_c16630	putative major facilitator superfamily (MFS) transporter	0	AAur_1759
ARUE_c16680	putative major facilitator superfamily (MFS) transporter	0	AAur_1763
ARUE_c16900	TrkA1:Trk system potassium uptake protein TrkA	1.00E-137	AAur_1784
ARUE_c16910	TrkA2:Trk system potassium uptake protein TrkA	1.00E-125	AAur_1786
ARUE_c16920	YdaO:uncharacterized amino acid permease YdaO	0	AAur_1787
ARUE_c17140	YdcO:inner membrane protein YdcO	0	AAur_1809
ARUE_c17160	YfnA1:uncharacterized amino acid permease YfnA	0	AAur_1811
ARUE_c18160	amino acid permease-associated region	2.00E-85	AAur_pTC20216
ARUE_c20200	RbsC3:ribose transport system permease protein RbsC	0	AAur_1888
ARUE_c20210	RbsB3:D-ribose-binding protein RbsB (3.6.3.17)	0	AAur_1889
ARUE_c20240	RbsA3:ribose import ATP-binding protein RbsA (3.6.3.17)	0	AAur_1892
ARUE_c20380	KdgT:2-keto-3-deoxygluconate permease	0	AAur_1906
ARUE_c20480	ABC transporter, ATP-binding protein	0	AAur_1915
ARUE_c20490	putative ABC transporter permease protein	0	AAur_1916
ARUE_c20500	extracellular solute-binding domain protein	0	AAur_1917
ARUE_c20520	MFS superfamily transporter	0	AAur_1919
ARUE_c20850	putative ABC-type dipeptide transport system	0	AAur_1953
ARUE_c20860	putative peptide permease protein	0	AAur_1954
ARUE_c20870	DppC1:dipeptide transport system permease protein DppC	1.00E-172	AAur_1955
ARUE_c20880	putative peptide ABC transporter ATP-binding protein y4tR	2.00E-179	AAur_1956
ARUE_c20890	AppF2:oligopeptide transport ATP-binding proteinAppF	0	AAur_1957

ARUE_c20930	putative ABC transporter, ATP-binding protein	1.00E-126	AAur_1961
ARUE_c20940	MntB1:ABC-type Mn2+ transport systems, permease component	2.00E-172	AAur_1962
ARUE_c20960	FimA:manganese ABC transporter substrate-binding lipoprotein FimA	2.00E-167	AAur_1964
ARUE_c21220	DdpA:putative D,D-dipeptide-binding extracellular protein DdpA	0	AAur_1969
ARUE_c21230	putative peptide transport system permease protein	4.00E-166	AAur_1970
ARUE_c21240	DppC2:dipeptide transport system permease protein DppC	4.00E-154	AAur_1971
ARUE_c21250	YejF:uncharacterized ABC transporter ATP-binding protein YejF	0	AAur_1972
ARUE_c21410	putative ABC-type sugar transport system	0	AAur_1988
ARUE_c21420	YphD:putative ABC transporter permease protein YphD	0	AAur_1989
ARUE_c21430	putative ribose/galactose/methyl galactoside import ATP-binding protein 2 (3.6.3.17)	3.00E-176	AAur_1990
ARUE_c21540	YfnA2:uncharacterized amino acid permease YfnA	0	AAur_2001
ARUE_c21590	putative Na+/H+ antiporter	0	AAur_2006
ARUE_c21680	Fe3+-siderophore ABC transporter, ATP-binding protein	1.00E-153	AAur_2015
ARUE_c21690	Fe3+-siderophore binding protein, FhuD family	0	AAur_2016
ARUE_c21700	Fe3+-hydroxamate ABC transporter, permease subunit	0	AAur_2017
ARUE_c21790	putative major facilitator superfamily (MFS) transporter	0	AAur_2026
ARUE_c21970	putative amino acid permease	0	AAur_2043
ARUE_c22050	putative permease component of ABC transport system	3.00E-158	AAur_2051
ARUE_c22060	ATPase component of putative multidrug ABC-type transport system; BcrA-like	0	AAur_2052
ARUE_c22150	Pit2:putative low-affinity inorganic phosphate transporter Pit	0	AAur_2060
ARUE_c22290	putative major facilitator superfamily (MFS) transporter	0	AAur_2074
ARUE_c22570	putative ABC transporter protein	6.00E-140	AAur_2101
ARUE_c22580	NodI3:Nod factor export ATP-binding protein I	2.00E-180	AAur_2102
ARUE_c22600	SufB:FeS cluster assembly protein SufB	0	AAur_2104
ARUE_c22610	SufD:putative FeS assembly protein SufD	0	AAur_2105
ARUE_c22630	SufC:putative FeS assembly ATPase SufC	9.00E-147	AAur_2107
ARUE_c22680	putative cobalt transport protein	6.00E-117	AAur_2111
ARUE_c22690	putative ABC transporter ATP-binding protein	1.00E-134	AAur_2113
ARUE_c22710	YdiF:uncharacterized ABC transporter ATP-binding protein YdiF	0	AAur_2115
ARUE_c22780	RbsA4:ribose import ATP-binding protein RbsA (3.6.3.17)	0	AAur_2122
ARUE_c22810	YlmA:uncharacterized ABC transporter ATP-binding protein YlmA	5.00E-153	AAur_2125

ARUE_c23100	putative glycosyl transferase / polysaccharide biosynthesis domain protein	0	AAur_2155
ARUE_c23390	TatA:twin-arginine translocation protein, TatA/E	1.00E-45	AAur_2184
ARUE_c23400	TatC:Sec-independent protein translocase protein TatC	3.00E-153	AAur_2185
ARUE_c24030	GlpF:glycerol uptake facilitator protein GlpF	5.00E-145	AAur_2248
ARUE_c24090	PcaK:4-hydroxybenzoate transporter PcaK	0	AAur_2254
ARUE_c24500	SecD:protein-export membrane protein SecD	0	AAur_2295
ARUE_c24830	YfiL:uncharacterized ABC transporter ATP-binding protein YfiL	0	AAur_2328
ARUE_c24840	putative ABC transporter, integral membrane protein	5.00E-158	AAur_2329
ARUE_c25050	putative ABC transporter permease protein ORF1	2.00E-162	AAur_2350
ARUE_c25060	AmyD:putative ABC transporter permease protein AmyD	1.00E-159	AAur_2351
ARUE_c25070	extracellular solute-binding domain protein	0	AAur_2352
ARUE_c25470	MscS:putative mechanosensitive ion channel protein MscS	1.00E-126	AAur_2391
ARUE_c25510	ABC transporter ATP-binding protein	0	AAur_2395
ARUE_c25810	ShiA6:shikimate transporter ShiA	0	AAur_2426
ARUE_c26130	Ffh:signal recognition particle protein Ffh	0	AAur_2458
ARUE_c26160	Amt1:putative ammonia channel	0	AAur_2461
ARUE_c26170	putative transmembrane efflux protein (MFS)	0	AAur_2462
ARUE_c26180	FtsY:cell division protein FtsY	0	AAur_2463
ARUE_c26220	putative major facilitator superfamily (MFS) transporter	0	AAur_2467
ARUE_c26560	ATPase component of ABC-type antimicrobial peptide transport system; BceA-like	7.00E-148	AAur_2501
ARUE_c26570	permease protein of ABC transporter	0	AAur_2502
ARUE_c26590	ATPase component of ABC-type transport system	4.00E-140	AAur_2504
ARUE_c26740	HbpA:heme-binding protein A	0	AAur_2519
ARUE_c26760	putative cation/multidrug efflux pump, AcrB family	0	AAur_2521
ARUE_c26820	putative anion permease, ArsB/NhaD superfamily	0	AAur_2526
ARUE_c26920	AroP:aromatic amino acid transport protein AroP	0	AAur_2537
ARUE_c26990	YhjE4:inner membrane metabolite transport protein YhjE	0	AAur_2544
ARUE_c27020	putative amino acid permease	0	AAur_2547
ARUE_c27050	ABC transporter ATP-binding protein	5.00E-160	AAur_2550
ARUE_c27060	SsuC2:putative aliphatic sulfonates transport permease protein SsuC	8.00E-137	AAur_2551
ARUE_c27070	putative ABC-type nitrate/sulfonate/bicarbonate transport system, permease component	2.00E-140	AAur_2552

ARUE_c27080	putative sulfonate ABC transporter, solute- binding protein	0	AAur_2553
ARUE_c27110	putative major facilitator superfamily (MFS) transporter	0	AAur_2555
ARUE_c27140	AqpZ:aquaporin Z	6.00E-173	AAur_2559
ARUE_c27210	YhcG2:uncharacterized ABC transporter ATP-binding protein YhcG	3.00E-172	AAur_2566
ARUE_c27350	putative integral membrane transport protein	0	AAur_2580
ARUE_c27360	NodI4:Nod factor export ATP-binding protein I	7.00E-176	AAur_2581
ARUE_c27500	AtpD:ATP synthase subunit beta (3.6.3.14)	0	AAur_2594
ARUE_c27510	AtpG:ATP synthase gamma chain	6.00E-161	AAur_2595
ARUE_c27520	AtpA:ATP synthase subunit alpha (3.6.3.14)	0	AAur_2596
ARUE_c27540	AtpF:ATP synthase subunit b	6.00E-96	AAur_2598
ARUE_c27550	ATP synthase F0, C subunit (3.6.3.14)	5.00E-35	AAur_2599
ARUE_c27560	AtpB:ATP synthase subunit a	5.00E-149	AAur_2600
ARUE_c27870	putative ABC-type Mn2+/Zn2+/Fe2+ transport system, permease component	4.00E-158	AAur_2629
ARUE_c27880	MntB2:manganese transport system ATP-binding protein MntB	5.00E-154	AAur_2631
ARUE_c27890	putative solute binding protein	0	AAur_2632
ARUE_c28060	EmrB/QacA subfamily drug resistance transporter	0	AAur_2649
ARUE_c28110	FtsX:cell division protein FtsX	5.00E-168	AAur_2655
ARUE_c28120	FtsE:cell division ATP-binding protein FtsE	2.00E-155	AAur_2656
ARUE_c28290	RfbB:O-antigen export system ATP-binding protein RfbB	2.00E-135	AAur_2673
ARUE_c28300	RfbA:O-antigen export system permease protein RfbA	6.00E-163	AAur_2674
ARUE_c28600	GsiA2:glutathione import ATP-binding protein GsiA	0	AAur_2706
ARUE_c28610	GsiD2:glutathione transport system permease protein GsiD	7.00E-170	AAur_2707
ARUE_c28620	DppB1:dipeptide transport system permease protein DppB	0	AAur_2708
ARUE_c28630	DppA:dipeptide transport protein DppA	0	AAur_2709
ARUE_c28740	GnuT:gluconate permease GnuT	0	AAur_2720
ARUE_c28960	putative amino acid ABC transporter	0	AAur_2743
ARUE_c28970	ArtQ2:arginine transport system permease proteinArtQ	8.00E-140	AAur_2744
ARUE_c28980	TcyC2:L-cystine import ATP-binding protein TcyC	2.00E-135	AAur_2745
ARUE_c29280	putative magnesium transporter MgtE (contains CBS domain)	0	AAur_2775
ARUE_c29310	putative twin-arginine translocation protein	9.00E-69	AAur_2778
ARUE_c29380	GluA:glutamate transport ATP-binding protein GluA	1.00E-139	AAur_2785
ARUE_c29390	GluB:glutamate-binding protein	2.00E-165	AAur_2786
ARUE_c29400	GlnM1:putative glutamine ABC transporter permease protein GlnM	5.00E-116	AAur_2787

ARUE_c29410	GluD:glutamate transport system permease protein GluD	5.00E-147	AAur_2788
ARUE_c29540	GsiA3:glutathione import ATP-binding protein GsiA	0	AAur_2801
ARUE_c29550	OppC:oligopeptide transport system permease protein OppC	0	AAur_2802
ARUE_c29560	DppB2:dipeptide transport system permease protein DppB	5.00E-171	AAur_2803
ARUE_c29580	OppA:oligopeptide-binding protein OppA	0	AAur_2804
ARUE_c29680	ProX:glycine betaine/choline-binding protein of an ABC-type transport system	2.00E-178	AAur_2814
ARUE_c29690	ProZ:ABC-type proline/glycine betaine transport systems, permease component	1.00E-133	AAur_2815
ARUE_c29700	ProW:ABC-type proline/glycine betaine transport systems, permease component	5.00E-132	AAur_2816
ARUE_c29710	OpuCA2:glycine betaine/carnitine/choline transport ATP- binding protein OpuCA	0	AAur_2817
ARUE_c29720	GTPase, YjeQ/EngC-like	0	AAur_2818
ARUE_c29800	YnfM:inner membrane transport protein YnfM	0	AAur_2826
ARUE_c29830	putative major facilitator superfamily (MFS) transporter	0	AAur_2829
ARUE_c29860	putative CorA-like metal ion (Mg2+/Co2+) transporter	0	AAur_2832
ARUE_c30130	PTS system, IIA component	3.00E-87	AAur_2860
ARUE_c30190	MsbA2:lipid A export ATP-binding/permease protein MsbA	0	AAur_2866
ARUE_c30730	UgpA2:sn-glycerol-3-phosphate transport system permease protein UgpA	0	AAur_2924
ARUE_c30740	UgpE:sn-glycerol-3-phosphate transport system permease protein UgpE	6.00E-161	AAur_2925
ARUE_c30750	UgpB:sn-glycerol-3-phosphate-binding extracellular protein UgpB	0	AAur_2926
ARUE_c30780	SecY:preprotein translocase subunit SecY	0	AAur_2929
ARUE_c31480	YbjJ1:inner membrane protein YbjJ	0	AAur_3000
ARUE_c31540	TauA2:putative ABC-type nitrate/sulfonate/bicarbonate transporter, substrate-binding protein TauA	0	AAur_3006
ARUE_c31550	TauB2:putative ABC-type nitrate/sulfonate/bicarbonate transporter, ATP-binding protein TauB	1.00E-166	AAur_3007
ARUE_c31560	TauC2:putative ABC-type nitrate/sulfonate/bicarbonate transporter, permease protein TauC	2.00E-167	AAur_3008
ARUE_c31600	MsmG:multiple sugar-binding transport system permease protein MsmG	2.00E-171	AAur_3012
ARUE_c31610	MsmF:multiple sugar-binding transport system permease protein MsmF	9.00E-166	AAur_3013
ARUE_c31620	extracellular solute-binding domain protein	0	AAur_3014
ARUE_c31640	Na+/H+ antiporter	0	AAur_3016

ARUE_c31760	putative amino acid permease	0	AAur_3027
ARUE_c31790	putative solute-binding lipoprotein	0	AAur_3030
ARUE_c31800	LacF2:lactose transport system permease protein LacF	6.00E-176	AAur_3031
ARUE_c31810	AraQ3:L-arabinose transport system permease protein AraQ	1.00E-172	AAur_3032
ARUE_c31830	putative major facilitator superfamily (MFS) transporter	0	AAur_3034
ARUE_c31900	ATPase component of putative ABC-type transport system	3.00E-180	AAur_3041
ARUE_c31910	putative permease protein of ABC-type transport system	4.00E-153	AAur_3042
ARUE_c31960	NosF:copper transport ATP-binding protein NosF	0	AAur_3047
ARUE_c31970	putative ABC transporter, permease protein	5.00E-148	AAur_3049
ARUE_c32000	putative voltage-gated potassium channel protein	8.00E-143	AAur_3051
ARUE_c32050	major facilitator superfamily (MFS) transporter	0	AAur_3056
ARUE_c32100	YhdG2:uncharacterized amino acid permease YhdG	0	AAur_3061
ARUE_c32160	RarD:protein RarD	0	AAur_3067
ARUE_c32210	putative major facilitator superfamily (MFS) transporter	0	AAur_3072
ARUE_c32340	putative nitrate/sulfonate ABC transporter, permease protein	6.00E-176	AAur_3086
ARUE_c32350	putative nitrate/sulfonate ABC transporter, ATP binding protein	5.00E-136	AAur_3087
ARUE_c32360	putative ABC transporter, substrate-binding protein, aliphatic sulfonates family	0	AAur_3088
ARUE_c32570	GlnH:glutamine-binding extracellular protein GlnH	5.00E-146	AAur_3104
ARUE_c32580	GlnM2:putative glutamine ABC transporter permease protein GlnM	5.00E-145	AAur_3105
ARUE_c32590	GlnQ3:glutamine transport ATP-binding protein GlnQ	5.00E-148	AAur_3106
ARUE_c32690	putative ABC transporter permease protein ORF2	6.00E-161	AAur_3120
ARUE_c32700	YurN6:putative ABC transporter permease protein YurN	0	AAur_3121
ARUE_c32710	extracellular solute-binding domain protein	0	AAur_3122
ARUE_c32760	putative major facilitator superfamily (MFS) transporter	0	AAur_3127
ARUE_c32840	putative cobalt-zinc-cadmium efflux permease	0	AAur_3137
ARUE_c32850	putative lipoprotein	2.00E-87	AAur_3138
ARUE_c33890	putative binding protein	0	AAur_3251
ARUE_c33900	putative ABC transporter permease protein	0	AAur_3252
ARUE_c33910	YurM4:putative ABC transporter permease protein YurM	5.00E-158	AAur_3253
ARUE_c34010	AnsP1:L-asparagine permease AnsP	0	AAur_3263
ARUE_c34050	TlrC:tylosin resistance ATP-binding protein TlrC	0	AAur_3267
ARUE_c34080	permease component of ABC-type metal cation transporter	4.00E-147	AAur_3270

ARUE_c34090	ATPase component of ABC-type metal cation transporter	1.00E-137	AAur_3271
ARUE_c34100	metal-binding extracellular protein interacting with ABC transporter	2.00E-179	AAur_3272
ARUE_c34250	CcsA:cytochrome c biogenesis protein CcsA	0	AAur_3286
ARUE_c34410	putative transmembrane efflux protein (MFS)	0	AAur_3305
ARUE_c34430	YesQ3:putative ABC transporter permease protein YesQ	6.00E-172	AAur_3307
ARUE_c34440	YesP2:putative ABC transporter permease protein YesP	0	AAur_3308
ARUE_c34450	YesO3:putative ABC transporter substrate- bindingprotein YesO	0	AAur_3309
ARUE_c34470	YesO4:putative ABC transporter substrate- bindingprotein YesO	0	AAur_3311
ARUE_c34480	YesQ4:putative ABC transporter permease protein YesQ	1.00E-179	AAur_3312
ARUE_c34490	YesP3:putative ABC transporter permease protein YesP	0	AAur_3313
ARUE_c34640	TrkH-like potassium uptake protein	0	AAur_3328
ARUE_c34650	potassium transport system dinucleotide-binding component TrkA	4.00E-151	AAur_3329
ARUE_c34930	ATPase component of ABC-type antimicrobial peptide transport system; BceA-like	3.00E-143	AAur_3355
ARUE_c34940	putative permease protein of ABC-type transport system	0	AAur_3356
ARUE_c34970	YurN7:putative ABC transporter permease protein YurN	8.00E-166	AAur_3359
ARUE_c34980	YurM5:putative ABC transporter permease protein YurM	1.00E-172	AAur_3360
ARUE_c34990	putative extracellular sugar-binding protein	0	AAur_3361
ARUE_c35090	MmpL:putative membrane protein MmpL	0	AAur_3372
ARUE_c35140	YgaD2:putative multidrug export ATP-binding/permease protein YgaD	0	AAur_3378
ARUE_c35150	YknV:uncharacterized ABC transporter ATP-binding protein YknV	0	AAur_3379
ARUE_c35220	DctA2:C4-dicarboxylate transport protein DctA	0	AAur_3387
ARUE_c35250	PucI:putative allantoin permease PucI	0	AAur_3390
ARUE_c35330	PbuG:guanine/hypoxanthine permease PbuG	0	AAur_3397
ARUE_c35400	PbuX:xanthine permease PbuX	0	AAur_3403
ARUE_c35500	YyaJ:putative metabolite transport protein YyaJ	0	AAur_3413
ARUE_c35550	OpdE:transcription regulatory protein OpdE	0	AAur_3418
ARUE_c35560	Amt2:putative ammonia channel	0	AAur_3419
ARUE_c35580	IcfA:carbonic anhydrase IcfA (4.2.1.1)	0	AAur_3421
ARUE_c35670	YceI2:putative metabolite transport protein YceI	0	AAur_3430
ARUE_c35820	ABC transporter ATP-binding protein	0	AAur_3463
ARUE_c35830	ABC transporter ATP-binding protein	0	AAur_3464
ARUE_c36030	YdbJ:uncharacterized ABC transporter ATP-binding protein YdbJ	1.00E-129	AAur_3486

ARUE_c36150	putative ABC transporter permease protein	2.00E-159	AAur_3499
ARUE_c36160	putative ABC transporter permease protein	2.00E-172	AAur_3500
ARUE_c36170	putative ABC transporter-binding protein	0	AAur_3501
ARUE_c36450	YgaY:uncharacterized transporter YgaY	0	AAur_3519
ARUE_c36590	ModC:molybdenum import ATP-binding protein ModC (3.6.3.29)	0	AAur_3533
ARUE_c36600	ModB:molybdenum transport system permease protein ModB	5.00E-129	AAur_3534
ARUE_c36610	ModA:molybdate-binding protein	1.00E-146	AAur_3535
ARUE_c36700	putative small multidrug resistance protein	8.00E-53	AAur_3544
ARUE_c36710	putative small multidrug resistance protein	4.00E-63	AAur_3545
ARUE_c36750	putative major facilitator superfamily (MFS) transporter	0	AAur_3549
ARUE_c36800	KgtP4:alpha-ketoglutarate permease KgtP	0	AAur_3554
ARUE_c36810	YycB2:uncharacterized transporter YycB	0	AAur_3555
ARUE_c36920	NatA:ATP-binding transport protein NatA	0	AAur_3565
ARUE_c36930	putative membrane protein (DUF990 family)	8.00E-155	AAur_3566
ARUE_c36940	putative membrane protein (DUF990 family)	6.00E-149	AAur_3567
ARUE_c36980	putative transmembrane efflux protein (MFS)	0	AAur_3570
ARUE_c37170	KgtP5:alpha-ketoglutarate permease KgtP	0	AAur_3588
ARUE_c37480	putative cation-transporting ATPase	0	AAur_3604
ARUE_c37590	putative translocator protein, LysE family	1.00E-112	AAur_3625
ARUE_c37680	major intrinsic protein (MIP) superfamily protein	4.00E-134	AAur_3632
ARUE_c37720	putative membrane protein	0	AAur_3636
ARUE_c37790	RhtB:homoserine/homoserine lactone efflux protein	5.00E-116	AAur_3643
ARUE_c37830	putative transmembrane efflux protein (MFS)	0	AAur_3647
ARUE_c37910	YbgF:uncharacterized amino acid permease YbgF	0	AAur_3655
ARUE_c37960	putative sulfate transporter	0	AAur_3661
ARUE_c38060	putative cobalt transport protein	1.00E-146	AAur_3670
ARUE_c38070	YkoD:putative HMP/thiamine import ATP-binding protein YkoD	0	AAur_3672
ARUE_c38110	putative multisubunit Na+/H+ antiporter, MnhG subunit	8.00E-71	AAur_3676
ARUE_c38120	putative multisubunit Na+/H+ antiporter, MnhE subunit	1.00E-101	AAur_3678
ARUE_c38130	MrpD:Na(+)/H(+) antiporter subunit D	0	AAur_3679
ARUE_c38140	putative multisubunit Na+/H+ antiporter, MnhC subunit	3.00E-90	AAur_3680
ARUE_c38150	PhaAB:putative K(+)/H(+) antiporter subunit A/B	0	AAur_3681
ARUE_c38160	putative major facilitator superfamily (MFS) transporter	0	AAur_3682

ARUE_c38180	YhaU:K(+)/H(+) antiporter YhaU	0	AAur_3684
ARUE_c38270	putative transmembrane efflux protein	0	AAur_3694
ARUE_c38540	putative integral membrane permease	6.00E-167	AAur_3721
ARUE_c38570	putative ABC transporter, solute binding protein	0	AAur_3724
ARUE_c38660	putative ABC transporter, solute binding protein	0	AAur_3733
ARUE_c38670	TcyC3:L-cystine import ATP-binding protein TcyC	6.00E-141	AAur_3734
ARUE_c38680	YecS2:inner membrane amino-acid ABC transporter permease protein YecS	0	AAur_3735
ARUE_c38960	Fe3+-siderophore ABC transporter, ATP-binding protein	7.00E-153	AAur_3763
ARUE_c38970	Fe3+-siderophore ABC transporter, permease subunit	0	AAur_3764
ARUE_c38980	Fe3+-siderophore ABC transporter, permease subunit	0	AAur_3765
ARUE_c38990	Fe3+-siderophore binding protein, FepB family	0	AAur_3766
ARUE_c39170	putative integral membrane protein	0	AAur_3783
ARUE_c39290	AnsP2:L-asparagine permease	0	AAur_3795
ARUE_c39480	putative translocator protein, LysE family	8.00E-106	AAur_3817
ARUE_c39590	ProP4:proline/betaine transporter ProP	0	AAur_3823
ARUE_c39750	putative major facilitator superfamily (MFS) transporter	0	AAur_3841
ARUE_c39770	YusP:uncharacterized MFS-type transporter YusP	0	AAur_3843
ARUE_c39870	Gtr:glucose transport protein	0	AAur_3844
ARUE_c39880	YfmO:multidrug efflux protein YfmO	0	AAur_3845
ARUE_c39930	DrrA:daunorubicin/doxorubicin resistance ATP-binding protein DrrA	5.00E-140	AAur_3850
ARUE_c39940	putative ABC transporter, efflux permease protein	6.00E-140	AAur_3851
ARUE_c39950	CycA2:D-serine/D-alanine/glycine transporter CycA	0	AAur_3852
ARUE_c39970	Fe3+-siderophore ABC transporter, ATP-binding protein, CeuD-like	4.00E-141	AAur_3854
ARUE_c39980	Fe3+-siderophore ABC transporter, permease subunit, CeuC-like	1.00E-167	AAur_3855
ARUE_c39990	Fe3+-siderophore ABC transporter, permease subunit, CeuB/CeuC-like	2.00E-179	AAur_3856
ARUE_c40000	siderophore binding protein, CeuA-like	1.00E-175	AAur_3857
ARUE_c40020	MtlA:PTS system mannitol-specific EIICBA component (2.7.1.69)	0	AAur_3859
ARUE_c40040	PtsI:phosphoenolpyruvate-protein phosphotransferase PtsI (2.7.3.9)	0	AAur_3861
ARUE_c40050	HPr4:phosphocarrier protein HPr	6.00E-45	AAur_3862
ARUE_c40170	hypothetical protein	0	AAur_3875
ARUE_c40180	uncharacterized ABC transporter ATP-binding protein	0	AAur_3876
ARUE_c40190	putative peptide transport permease protein	0	AAur_3877
ARUE_c40200	putative peptide transport permease protein	0	AAur_3878

ARUE_c40310	YeeF:inner membrane transport protein YeeF	0	AAur_3887
ARUE_c40470	ProP5:proline/betaine transporter ProP	0	AAur_3902
ARUE_c40820	putative extracellular solute-binding lipoprotein	0	AAur_3941
ARUE_c40830	putative ABC-type sugar transport system, permease component	3.00E-177	AAur_3942
ARUE_c40840	AraQ6:L-arabinose transport system permease protein AraQ	5.00E-174	AAur_3943
ARUE_c40870	putative major facilitator superfamily (MFS) transporter	0	AAur_3946
ARUE_c40920	putative cytosine/purine/uracil/thiamine/allantoin permease family protein	0	AAur_3952
ARUE_c40990	putative amino acid permease	0	AAur_3959
ARUE_c41010	putative ZIP zinc transporter	4.00E-140	AAur_3961
ARUE_c41040	putative integral membrane protein (DMT superfamily)	6.00E-172	AAur_3965
ARUE_c41140	EmrB/QacA subfamily drug resistance transporter	0	AAur_3973
ARUE_c41220	BenK1:benzoate transport protein	0	AAur_3981
ARUE_c41260	putative integral membrane protein	4.00E-137	AAur_3985
ARUE_c41500	YxlA:putative purine-cytosine permease YxlA	0	AAur_4010
ARUE_c41530	MscL:large conductance mechanosensitive channelprotein MscL	6.00E-82	AAur_4013
ARUE_c41910	ShiA7:shikimate transporter ShiA	0	AAur_4048
ARUE_c42020	YgbN2:inner membrane permease YgbN	0	AAur_4059
ARUE_c42090	putative integral membrane permease	7.00E-168	AAur_4066
ARUE_c42220	AraQ7:L-arabinose transport system permease protein AraQ	4.00E-170	AAur_4079
ARUE_c42230	UgpA3:sn-glycerol-3-phosphate transport system permease protein UgpA	8.00E-179	AAur_4080
ARUE_c42240	putative extracellular solute-binding protein	0	AAur_4081
ARUE_c42350	putative ABC transporter permease protein y4oR	8.00E-166	AAur_4090
ARUE_c42360	putative ABC transporter permease protein	0	AAur_4091
ARUE_c42370	putative extracellular sugar-binding protein	0	AAur_4092
ARUE_c42400	putative heavy metal-translocating (Cu+/Ag+) P- type ATPase, CopA-like	0	AAur_4095
ARUE_c42430	putative integral membrane protein DUF6 domain	0	AAur_4098
ARUE_c42470	putative MATE efflux family protein	0	AAur_4102
ARUE_c42600	ShiA8:shikimate transporter ShiA	0	AAur_4132
ARUE_c42760	putative gluconate transporter	0	AAur_4146
ARUE_c42880	putative ABC transporter, ATP-binding protein	1.00E-122	AAur_4158
ARUE_c42990	GabP:GABA permease GabP	0	AAur_4169
ARUE_c43050	ATPase component of putative antimicrobial peptides ABC transporter; BcrA-like	7.00E-176	AAur_4175

ARUE_c43070	YbjJ2:inner membrane protein YbjJ	0	AAur_4177
ARUE_c43090	ABC transporter F family member	0	AAur_4180
ARUE_c43150	putative ABC transporter, permease protein	0	AAur_4186
ARUE_c43160	ATPase component of putative ABC transporter	6.00E-124	AAur_4188
ARUE_c43240	MviN:integral membrane protein MviN	0	AAur_4196
ARUE_c43360	OxaA:membrane protein OxaA	0	AAur_4207
ARUE_113p00100	putative MFS-family transporter	1.00E-19	AAur_1381
ARUE_232p00330	Major facilitator superfamily MFS_1	0	AAur_pTC20052
ARUE_232p00430	czcD: Cobalt-zinc-cadmium resistance protein CzcD	5.00E-153	AAur_pTC20044
ARUE_232p00610	Putative arsenite efflux pump	2.00E-158	AAur_pTC20051
ARUE_232p00710	ctpG: Probable cation-transporting ATPase G	0	AAur_pTC20031
ARUE_232p00780	copB: Probable copper-exporting P-type ATPase B	0	AAur_pTC20028

Table S2.2: Putative transporters and binding proteins of *Arthrobacter* sp. Rue61a unrelated to those of *A. aurescens* TC1.

**Locus_tag**	**Annotation**
ARUE_c00800	transmembrane transport protein
ARUE_c01680	PTS-dependent dihydroxyacetone kinase, dihydroxyacetone-binding subunit DhaK
ARUE_c01690	PTS-dependent dihydroxyacetone kinase, ADP-binding subunit DhaL
ARUE_c01700	dihydroxyacetone kinase, phosphotransfer subunit/phosphocarrier protein HPr
ARUE_c02370	putative ABC transporter substrate-bindingprotein YesO
ARUE_c03570	ABC-type Fe3+-hydroxamate transporter, extracellular iron-siderophore binding protein
ARUE_c03580	siderophore interacting protein
ARUE_c04060	major facilitator superfamily MFS_1
ARUE_c04580	putative metabolite transport protein YaaU
ARUE_c08460	phosphate transport system protein PhoU
ARUE_c15890	inner membrane transport protein YdhP
ARUE_c16740	ATP-binding/permease protein CydD
ARUE_c17270	putative nicotinamide mononucleotide transporterPnuC
ARUE_c17690	putative sugar ABC transporter, permease protein
ARUE_c17700	putative sugar ABC transporter, permease protein
ARUE_c17920	MFS-type transporter
ARUE_c18180	ribose import ATP-binding protein RbsA
ARUE_c18190	ribose transport system permease protein RbsC
ARUE_c18200	D-ribose-binding protein RbsB
ARUE_c18250	amino acid permease-associated region
ARUE_c18330	EmrB/QacA subfamily drug resistance transporter
ARUE_c18480	putative sugar ABC transporter, permease protein
ARUE_c18490	putative sugar ABC transporter, permease protein
ARUE_c18500	putative secreted sugar-binding protein
ARUE_c18510	ABC transporter
ARUE_c18530	sugar transporter
ARUE_c18570	putative glucitol transport protein GutA
ARUE_c18650	putative tartrate transporter TtuB
ARUE_c18680	major facilitator superfamily MFS_1
ARUE_c19080	proline/betaine transporter ProP
ARUE_c19180	TRAP C4-dicarboxylate transport system permease DctM subunit
ARUE_c19250	putative ABC transporter permease protein YesQ
ARUE_c19260	sn-glycerol-3-phosphate transport system permease protein UgpA
ARUE_c19290	putative ABC transporter substrate-binding protein
ARUE_c19300	sn-glycerol-3-phosphate import ATP-bindingprotein UgpC
ARUE_c19680	putative MFS transporter
ARUE_c19730	alpha-ketoglutarate permease KgtP
ARUE_c19810	uncharacterized transporter YycB
ARUE_c19850	binding-protein-dependent transport systems inner membrane component
ARUE_c19860	putative ABC transporter permease protein
ARUE_c19870	extracellular solute-binding protein, family 1
ARUE_c21050	shikimate transporter ShiA
ARUE_c21140	glutamine transport ATP-binding protein GlnQ
ARUE_c21150	inner membrane amino-acid ABC transporter permease protein YecS
ARUE_c21160	ABC-type amino acid transport/signal transduction system
ARUE_c24490	protein-export membrane protein SecF

ARUE_c24510	preprotein translocase, YajC subunit
ARUE_c26600	putative permease protein of ABC-type transport system
ARUE_c27400	putative permease
ARUE_c31260	MFS-type transporter
ARUE_c31300	putative manganese transport protein MntH
ARUE_c37340	ABC-type sugar transport system extracellular component
ARUE_c37350	ABC transporter permease protein
ARUE_c37360	L-arabinose transport system permease protein AraQ
ARUE_c37450	putative ABC transporter permease protein YurN
ARUE_c37460	L-arabinose transport system permease protein AraQ
ARUE_c38030	ABC transporter ATP-binding protein
ARUE_c39800	ABC transporter, substrate binding component
ARUE_c39810	proline/glycine/betaine transporer, permease component
ARUE_c39820	ABC transporter, permease component
ARUE_c39830	glycine betaine/carnitine/choline transport ATP-binding protein, OpuCA-like
ARUE_c41290	putative permease
ARUE_c41710	benzoate transport protein
ARUE_c43040	putative permease component of ABC-type transport system
ARUE_232p00230	major facilitator superfamily MFS_1
ARUE_232p00560	putative heavy metal-translocating P-type ATPase
ARUE_232p00580	putative divalent metal ion transporter
ARUE_232p00620	CBS-pair_CorC_Hly-domain- (transporter associated domain-) containing protein
ARUE_232p00630	CBS-pair_CorC_Hly-domain- (transporter associated domain-) containing protein
ARUE_232p01200	putative ABC transporter permease protein
ARUE_232p01210	putative ATP-binding protein
ARUE_232p01220	putative binding protein
ARUE_232p01490	amino-acid ABC transporterATP-binding protein YecC
ARUE_232p01500	amino-acid ABC transporter permease protein YckA1
ARUE_232p01510	amino-acid ABC transporter permease protein YckA2
ARUE_232p01520	amino acid ABC transporter, extracellular amino acid-binding protein
ARUE_232p01550	inner membrane metabolite transport protein YhjE
ARUE_232p01590	putative peptide transport system permease protein
ARUE_232p01600	oligopeptide transport system permease protein AppC
ARUE_232p01610	uncharacterized ABC transporter ATP-binding protein
ARUE_232p01670	putative metabolite transport protein YceI
ARUE_232p01720	putative potassium-transporting ATPase, A subunit
