# Supplementary material for: Complete genome sequence and metabolic potential of the quinaldine-degrading bacterium Arthrobacter sp. Rue61a
Source: BMC Genomics. 2012 Oct 6;13:534. doi: 10.1186/1471-2164-13-534 (PMC3534580; doi:10.1186/1471-2164-13-534)
Supplement: Additional file 3 — Tables S3. Genes of Arthrobacter sp. Rue61a, grouped according to functions, as discussed in the text. [file 1471-2164-13-534-S3.pdf]

Additional File 3:

Genes of *Arthrobacter* sp. Rue61a, grouped according to functions, as discussed in the text

---

Table S3.1: Genes of *Arthrobacter* sp. Rue61a presumed to be involved in osmoprotection.

Locus_tag	Gene symbol	Predicted protein product	EC #
ARUE_c02780	*proP1*	proline/betaine transporter, MF superfamily	
ARUE_c04460		betaine/carnitine/choline transporter, BCCT family	
ARUE_c04830		choline oxidase	1.1.3.17
ARUE_c04840	*gbsA*	betaine aldehyde dehydrogenase	1.2.1.8
ARUE_c05460	*proP2*	proline/betaine transporter, MF superfamily	
ARUE_c06090	*yehY*	ABC-type putative osmoprotectant uptake system, permease protein	
ARUE_c06100	*opuCA1*	ABC-type glycine betaine/carnitine/choline transport system, ATP-binding protein	
ARUE_c06110	*opuBB*	ABC-type glycine betaine/carnitine/choline transport system, permease protein	
ARUE_c06120	*osmF*	osmoprotectant uptake system, substrate-binding protein	
ARUE_c08620	*treS1*	trehalose synthase	5.4.99.16
ARUE_c08830	*otsB*	trehalose-6-phosphate phosphatase	3.1.3.12
ARUE_c08840	*otsA*	trehalose-6-phosphate synthase	2.4.1.15
ARUE_c16900	*trkA1*	Trk system potassium uptake protein, dinucleotide-binding regulatory component	
ARUE_c16910	*trkA2*	Trk system potassium uptake protein, dinucleotide-binding regulatory component	
ARUE_c17450	*gltB*	glutamate synthase [NADPH/NADH], alpha subunit	1.4.1.13 or 1.4.1.14
ARUE_c17460	*gltD*	glutamate synthase [NADPH/NADH], beta subunit	1.4.1.13 or 1.4.1.14
ARUE_c19080	*proP3*	proline/betaine transporter, MF superfamily	
ARUE_c25180	*proA*	gamma-glutamyl phosphate reductase	1.2.1.41
ARUE_c25190	*proB*	glutamate 5-kinase	2.7.2.11
ARUE_c25470	*mscS*	putative mechanosensitive ion channel protein	
ARUE_c29680	*proX*	glycine betaine/choline-binding protein of an ABC-type transport system	
ARUE_c29690	*proZ*	ABC-type proline/glycine betaine transport system, permease component	
ARUE_c29700	*proW*	ABC-type proline/glycine betaine transport system, permease component	
ARUE_c29710	*opuCA2*	glycine betaine/carnitine/choline transport system, OpuCA-like ATP-binding protein	
ARUE_c30460	*treZ*	malto-oligosyltrehalose trehalohydrolase	3.2.1.141
ARUE_c30470	*treY*	maltooligosyl trehalose synthase	5.4.99.15
ARUE_c34610	*gdh*	homooligomeric $NADP^+$-specific glutamate dehydrogenase	
ARUE_c34640	*trkH*	TrkH-like potassium uptake protein, ion-conducting transmembrane component	
ARUE_c34650	*trkA3*	Trk system potassium uptake protein, dinucleotide-binding regulatory component	
ARUE_c34660	*proC*	pyrroline-5-carboxylate reductase	1.5.1.2
ARUE_c39590	*proP4*	proline/betaine transporter, MF superfamily	
ARUE_c39800		ABC-type transporter, substrate binding component	

ARUE_c39810		ABC-type proline/betaine transporter, permease component	
ARUE_c39820		ABC-type transporter, permease component	
ARUE_c39830	*opuCA3*	glycine betaine/carnitine/choline transporter, OpuCA-like ATP-binding protein	
ARUE_c40470	*proP5*	proline/betaine transporter, MF superfamily	
ARUE_c41530	*mscL*	large conductance mechanosensitive ion channel protein	
ARUE_c42120	*treS2*	trehalose synthase	5.4.99.16
ARUE_232p00510	*trkH*	TrkH-type potassium uptake protein	

Table S3.2: Genes of *Arthrobacter* sp. Rue61a possibly involved in formation of reactive oxygen species and in protection against oxidative stress.

**Locus_tag**	**Gene symbol**	**Predicted protein product**	**EC #**
ARUE_c00400	*puo*	putrescine oxidase	1.4.3.10
ARUE_c00520		putative sugar alcohol dehydrogenase or oxidase/alditol oxidase	
ARUE_c01660		FAD/FMN-containing dehydrogenase or oxidase	
ARUE_c04530		N,N-dimethylglycine oxidase	1.5.3.10
ARUE_c04810		acyl-coenzyme A oxidase	1.3.3.6
ARUE_c04830		choline oxidase	1.1.3.17
ARUE_c04860		putative molybdopterin oxidoreductase, sulfite oxidase family	
ARUE_c05480		Cu^{2+}-containing amine oxidase	
ARUE_c06000	*ydbD*	putative Mn catalase	1.11.1.6
ARUE_c06400	*gpx*	glutathione peroxidase	1.11.1.9
ARUE_c07090		acyl-coenzyme A dehydrogenase/oxidase family protein	
ARUE_c09150		acyl-coenzyme A dehydrogenase/oxidase family protein	
ARUE_c09600		putative multicopper oxidase family protein	
ARUE_c11380	*soxA1*	putative monomeric sarcosine oxidase	1.5.3.1
ARUE_c11870		OsmC family stress protein or peroxiredoxin	
ARUE_c14160		acyl-coenzyme A dehydrogenase/oxidase family protein	
ARUE_c14890		putative molybdopterin oxidoreductase, sulfite oxidase family	
ARUE_c16720–16730		putative cytochrome d ubiquinol oxidase	1.10.2.-
ARUE_c17720		pyranose 2-oxidase	1.1.3.10
ARUE_c17930		pyranose 2-oxidase	1.1.3.10
ARUE_c19520		pyridoxine 5'-phosphate oxidase-like protein	
ARUE_c19630		putative multicopper oxidase family protein	
ARUE_c19980	*katE*	catalase	1.11.1.6
ARUE_c21130	*soxA2*	putative monomeric sarcosine oxidase oxidase	
ARUE_c21770		putative uncharacterized peroxidase	
ARUE_c22440	*sodA*	superoxide dismutase	1.15.1.1
ARUE_c22940		acyl-coenzyme A dehydrogenase/oxidase family protein	
ARUE_c23620	*ctaE*	putative cytochrome oxidase subunit III	1.9.3.1
ARUE_c25860		putative peroxiredoxin, AhpE-like	1.11.1.15
ARUE_c26720		putative peroxiredoxin (PRX), BCP subfamily	1.11.1.15
ARUE_c26880	*nadB*	L-aspartate oxidase	1.4.3.16
ARUE_c29070	*hemG*	protoporphyrinogen oxidase	1.3.3.4
ARUE_c29170	*thiO*	putative glycine oxidase/D-amino acid oxidase	
ARUE_c32080	*katA*	catalase	1.11.1.6
ARUE_c35300–35310		putative xanthine dehydrogenase/xanthine oxidase	1.17.1.4/ 1.17.3.2
ARUE_c35390	*uox*	urate oxidase	1.7.3.3
ARUE_c36380		putative peroxiredoxin (PRX), BCP subfamily	1.11.1.15
ARUE_c36580		pyridoxine 5'-phosphate oxidase family protein	
ARUE_c36630		FAD/FMN-containing dehydrogenase or oxidase	
ARUE_c36760	*soxR*	redox-sensitive transcription factor SoxR	
ARUE_c37540		alkylhydroperoxidase like protein, AhpD family	
ARUE_c37570		Cu^{2+}-containing primary amine oxidase	1.4.3.4
ARUE_c39110		putative sulfite oxidase	1.8.3.1
ARUE_c39620–39650	*soxBDAG*	sarcosine oxidase	1.5.3.1
ARUE_c41520	*xyoA*	putative xylitol oxidase	1.1.3.41
ARUE_232p00320		putative multicopper oxidase family protein	
ARUE_232p00590		multicopper oxidase	

ARUE_232p01420	*thiO/thiG*	bifunctional protein ThiO/ThiG (ThiG, amino acid oxidase)	
ARUE_113p00040–00060	*meqABC*	quinaldine 4-oxidase	
ARUE_113p00140		putative Fe/Mn superoxide dismutase	1.15.1.1

Table S3.3: Genes of *Arthrobacter* sp. Rue61a presumed to be involved in acquisition of iron.

**Locus_tag**	**Gene symbol**	**Predicted protein product**	**EC #**
ARUE_c02640	*rhbB*	L-2,4-diaminobutyrate decarboxylase	4.1.1.86
ARUE_c02650		putative lysine/ornithine N-monooxygenase	
ARUE_c02660		putative acetyltransferase	
ARUE_c02670	*rhbF*	rhizobactin siderophore biosynthesis protein	
ARUE_c03570		ABC-type Fe^{3+}-hydroxamate transporter, extracellular iron-siderophore binding protein	
ARUE_c03580		siderophore interacting protein with a ferredoxin reductase-like (FNR-like) domain structure	
ARUE_c03590		Fe^{3+}-siderophore ABC transporter, transmembrane subunit	
ARUE_c03600		Fe^{3+}-siderophore ABC transporter, transmembrane subunit	
ARUE_c03610		Fe^{3+}-siderophore ABC transporter, ATP-binding protein	
ARUE_c05260		Fe^{3+}-siderophore ABC transporter, ATP-binding protein	
ARUE_c05270		Fe^{3+}-siderophore ABC transporter, transmembrane subunit	
ARUE_c05280		Fe^{3+}-siderophore ABC transporter, transmembrane subunit	
ARUE_c05290		Fe^{3+}-siderophore binding protein, FhuD family	
ARUE_c07040		putative Fe^{3+} transport system, SfuC-like ATP binding protein	3.6.3.30
ARUE_c07050		putative Fe^{3+}-transport system, SfuB-like permease protein	3.6.3.30
ARUE_c07060		putative Fe^{3+}-binding extracellular protein, SfuA-/ FbpA-like	3.6.3.30
ARUE_c09470		siderophore interacting protein with a ferredoxin reductase-like (FNR-like) domain structure	
ARUE_c21680		Fe^{3+}-siderophore ABC transporter, ATP-binding protein	
ARUE_c21690		Fe^{3+}-siderophore binding protein, FhuD family	
ARUE_c21700		Fe^{3+}-hydroxamate ABC transporter, permease subunit	
ARUE_c27860	*fur1*	ferric uptake regulation protein Fur	
ARUE_c32070	*fur2*	ferric uptake regulation protein Fur	
ARUE_c38950		siderophore interacting protein with a ferredoxin reductase-like (FNR-like) domain structure	
ARUE_c38960		Fe^{3+}-siderophore ABC transporter, ATP-binding protein	
ARUE_c38970		Fe^{3+}-siderophore ABC transporter, permease subunit	
ARUE_c38980		Fe^{3+}-siderophore ABC transporter, permease subunit	
ARUE_c38990		Fe^{3+}-siderophore binding protein, FepB family	
ARUE_c39970		Fe^{3+}-siderophore ABC transporter, ATP-binding protein, CeuD family	
ARUE_c39980		Fe^{3+}-siderophore ABC transporter, permease subunit, CeuC family	
ARUE_c39990		Fe^{3+}-siderophore ABC transporter, permease subunit, CeuB family	
ARUE_c40000		siderophore binding protein (FatB), CeuA family	

Table S3.4: Genes of *Arthrobacter* sp. Rue61a presumed to be involved in response to heavy metal ions.

**Locus_tag**	**Gene symbol**	**Predicted protein product**	**EC #**
ARUE_c00900		putative NAD(P)H:flavin oxidoreductase/NAD(P)H nitroreductase	
ARUE_c03850	*czcD*	putative cation (cobalt-zinc-cadmium) efflux transporter	
ARUE_c19540		putative heavy-metal-translocating P-type ATPase	
ARUE_c26820		putative anion permease, ArsB/NhaD superfamily	
ARUE_c33070		putative NAD(P)H:flavin oxidoreductase/NAD(P)H nitroreductase	
ARUE_c37620	*arsC1*	putative arsenate reductase ArsC	1.20.4.1
ARUE_c37630	*arsC2*	putative arsenate reductase ArsC	1.20.4.1
ARUE_c37660	*arsC3*	putative arsenate reductase ArsC	1.20.4.1
ARUE_c41610		possible chromate reductase; YieF/ChrR-like NAD(P)H-dependent FMN reductase	
ARUE_c42390		heavy-metal-associated domain (HMA) -containing protein; possible copper chaperone	
ARUE_c42400	*copA*	putative heavy metal-translocating (Cu^{+}/Ag^{+}) P-type ATPase, CopA-like	
ARUE_232p00330		putative major facilitator superfamily transporter; NreB-like	
ARUE_232p00400	*copC*	putative copper resistance protein CopC	
ARUE_232p00430	*czcD*	cation (cobalt-zinc-cadmium) efflux transporter CzcD	
ARUE_232p00560		putative Cd^{2+}/Zn^{2+}/Pb^{2+}-translocating P-type ATPase	
ARUE_232p00610		putative ACR3-type arsenite efflux pump	
ARUE_232p00710		putative Cd^{2+}/Zn^{2+}/Pb^{2+}-translocating P-type ATPase	
ARUE_232p00780		putative copper-translocating P-type ATPase	
ARUE_232p00800	*merA*	putative mercuric reductase MerA	1.16.1.1

Table S3.5: Genes of *Arthrobacter* sp. Rue61a possibly involved in resistance to bacitracin.

**Locus_tag**	**Predicted protein product**
ARUE_c22050	putative permease component of ABC transport system
ARUE_c22060	ATPase component of putative ABC-type peptide antibiotic transport system; BcrA subfamily
ARUE_c26540	putative sensor histidine kinase
ARUE_c26550	putative response regulator responding to cell wall stress/antimicrobial peptides
ARUE_c26560	ATPase component of ABC-type antimicrobial peptide transport system; BceA-like
ARUE_c26570	permease protein of ABC transporter
ARUE_c34930	ATPase component of ABC-type antimicrobial peptide transport system; BceA-like
ARUE_c34940	putative permease protein of ABC-type transport system
ARUE_c43020	putative response regulator containing a CheY-like signal receiver domain and LuxR-C-like HTH DNA-binding domain
ARUE_c43030	putative sensor histidine kinase
ARUE_c43040	putative permease component of ABC-type transport system
ARUE_c43050	ATPase component of putative antimicrobial peptides ABC transporter; BcrA-like
ARUE_c43130	putative response regulator containing a CheY-like signal receiver domain and LuxR-C-like HTH DNA-binding domain
ARUE_c43140	putative signal transduction histidine kinase
ARUE_c43150	putative ABC transporter, permease protein
ARUE_c43160	ATPase component of putative ABC transporter

Table S3.6: Genes of *Arthrobacter* sp. Rue61a involved in the metabolism of aromatic and N-heterocyclic compounds (see also Additional File 4 for Figures of pathways).

**Locus_tag**	**Gene symbol**	**Predicted protein product**	**EC #**
ARUE_c02440	*styA2B*	putative styrene monooxygenase, StyA2B-like	
ARUE_c02450	*styA1*	putative oxygenase component, StyA1-like	
ARUE_c05840	*hpaC*	flavin reductase; putative reductase component of 4-hydroxyphenylacetate 3-monooxygenase	
ARUE_c05860	*hpaB*	putative 4-hydroxyphenylacetate 3-monooxygenase oxygenase component (HpaB)	1.14.14.-
ARUE_c24100	*vanB*	VanB-like reductase component (flavin-iron/sulfur protein) of oxygenase system	
ARUE_c24110	*vanA*	vanillate O-demethylase oxygenase component VanA	1.14.13.82
ARUE_c33590	*paaG*	putative ring 1,2-epoxyphenylacetyl-CoA isomerase, oxepin-CoA-forming	
ARUE_c33600	*paaE*	ring 1,2-phenylacetyl-CoA epoxidase component	
ARUE_c33610	*paaD*	ring 1,2-phenylacetyl-CoA epoxidase component	
ARUE_c33620	*paaC*	ring 1,2-phenylacetyl-CoA epoxidase component	
ARUE_c33630	*paaB*	ring 1,2-phenylacetyl-CoA epoxidase component	
ARUE_c33640	*paaA*	ring 1,2-phenylacetyl-CoA epoxidase component	
ARUE_c33800	*paaI*	phenylacetic acid degradation protein, thioesterase PaaI	
ARUE_c33810	*paaK*	phenylacetate-coenzyme A ligase	
ARUE_c40570	*hpcH*	4-hydroxy-2-oxo-heptane-1,7-dioate aldolase HpcH	4.1.2.-
ARUE_c40580	*hpcG*	2-oxo-hepta-3-ene-1,7-dioic acid hydratase HpcG	4.2.1.-
ARUE_c40590	*hpcB*	3,4-dihydroxyphenylacetate 2,3-dioxygenase HpcB	1.13.11.2
ARUE_c40600	*hpcC*	5-carboxymethyl-2-hydroxymuconate semialdehyde dehydrogenase HpcC	1.2.1.60
ARUE_c40620	*hpaG*	4-hydroxyphenylacetate degradation bifunctional isomerase/decarboxylase HpaG	4.1.1.67, 5.3.3.-
ARUE_c41200	*pobA*	4-hydroxybenzoate hydroxylase PobA	1.14.13.2
ARUE_c41720		putative 2-aminobenzoyl-CoA monooxygenase/ reductase	1.14.13.40
ARUE_c41730		anthranilate-CoA ligase	6.2.1.32
ARUE_c41790	*pcaR*	Pca regulon regulatory protein PcaR	
ARUE_c41800	*pcaJ*	3-oxoadipate CoA-transferase subunit B	2.8.3.6
ARUE_c41810	*pcaI*	3-oxoadipate CoA-transferase subunit A	2.8.3.6
ARUE_c41820	*pcaF*	beta-ketoadipyl-CoA thiolase PcaF	2.3.1.174
ARUE_c41830	*pcaC*	4-carboxymuconolactone decarboxylase PcaC	4.1.1.44
ARUE_c41840	*pcaD*	3-oxoadipate enol lactone hydrolase PcaD	3.1.1.24
ARUE_c41850	*pcaB*	3-carboxy-cis,cis-muconate cycloisomerase PcaB	5.5.1.2
ARUE_c41860	*pcaG*	protocatechuate 3,4-dioxygenase alpha chain	1.13.11.3
ARUE_c41870	*pcaH*	protocatechuate 3,4-dioxygenase beta chain	1.13.11.3
ARUE_c41880	*hpd*	4-hydroxyphenylpyruvate dioxygenase Hpd	1.13.11.27
ARUE_113p00030		putative XdhC protein	
ARUE_113p00040	*meqA*	quinaldine 4-oxidase, small subunit	1.3.99.-
ARUE_113p00050	*meqB*	quinaldine 4-oxidase, medium subunit	1.3.99.-
ARUE_113p00060	*meqC*	quinaldine 4-oxidase, large subunit	1.3.99.-
ARUE_113p00070	*meqD*	1*H*-4-oxoquinaldine 3-monooxygenase	1.14.13.-
ARUE_113p00080	*meqE*	1*H*-3-hydroxy-4-oxoquinaldine 2,4-dioxygenase	1.13.11.48
ARUE_113p00090	*meqF*	N-acetylanthranilate amidase	3.5.1.13
ARUE_113p00160	*meqR1*	transcriptional regulator	
ARUE_113p00210		putative acyl-CoA thioesterase	3.1.2.-
ARUE_113p00220		anthranilate CoA ligase	6.2.1.32
ARUE_113p00230		putative 2-aminobenzoyl-CoA monooxygenase/ reductase	1.14.13.40
ARUE_113p00240	*meqR2*	transcriptional regulator	
