# Supplementary material for: Complete genome sequence and metabolic potential of the quinaldine-degrading bacterium Arthrobacter sp. Rue61a
Source: BMC Genomics. 2012 Oct 6;13:534. doi: 10.1186/1471-2164-13-534 (PMC3534580; doi:10.1186/1471-2164-13-534)

Additional File 4:

Biodegradation pathways of *Arthrobacter* sp. Rue61a

Degradation of quinaldine:

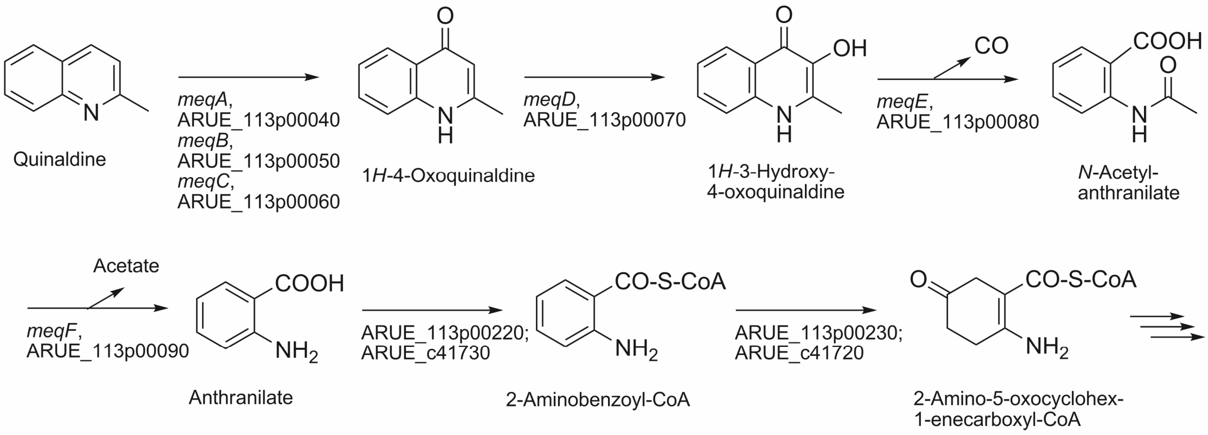

Degradation of 4-hydroxybenzoate, vanillate, and protocatechuate via the *ortho* pathway:

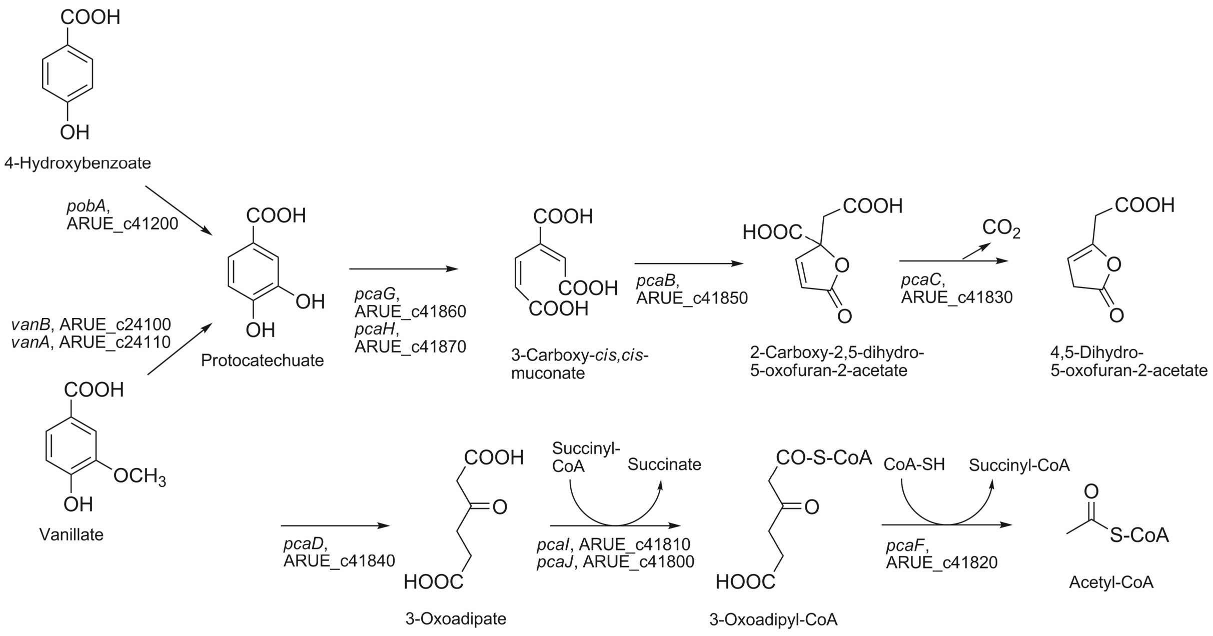

Degradation of 4-hydroxyphenylacetate and homoprotocatechuate via the *meta* pathway:

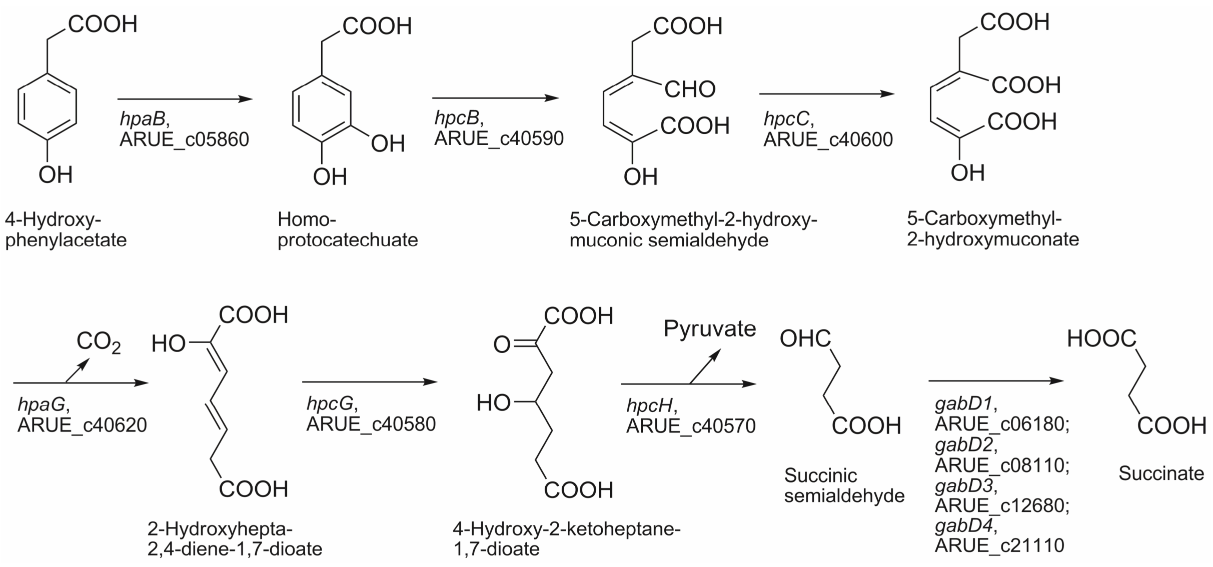

Choline, creatine and sarcosine metabolism:

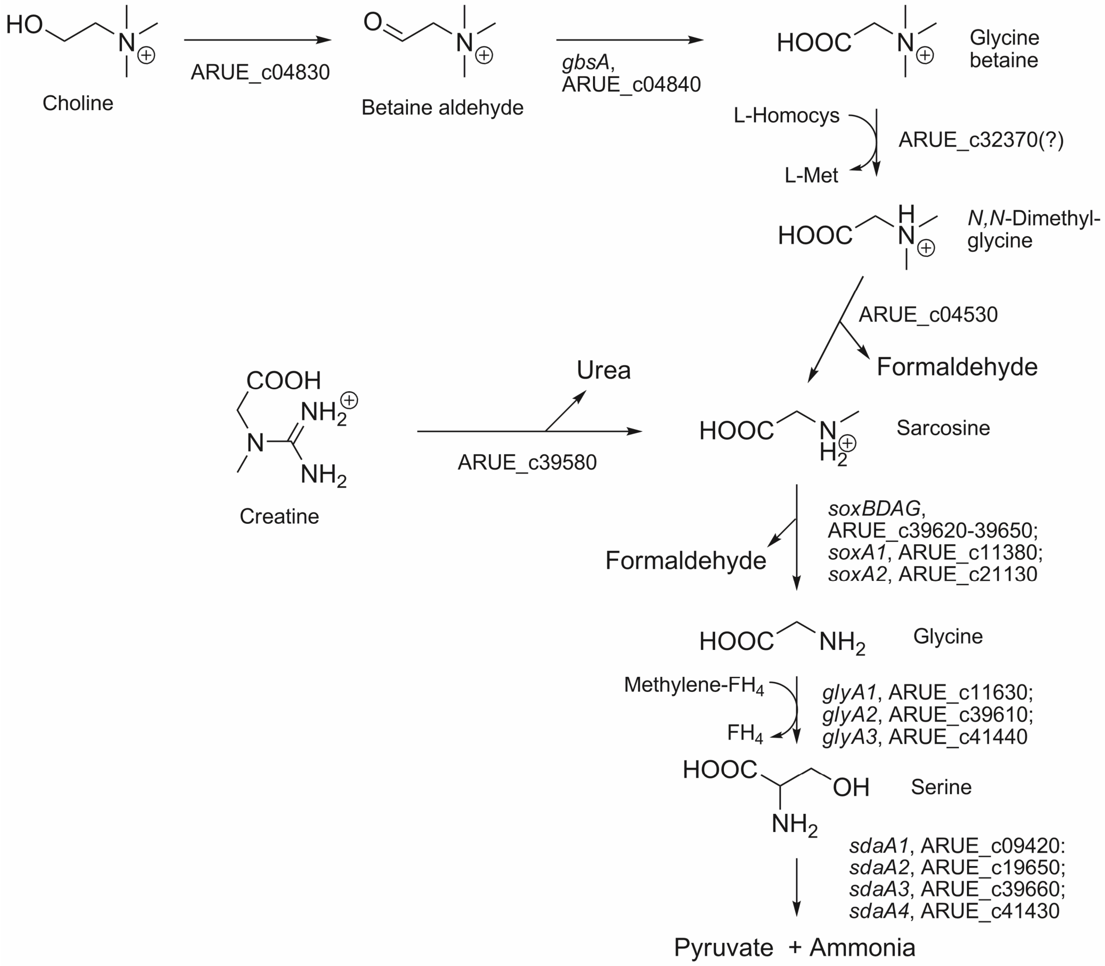

Agmatine and putrescine degradation:

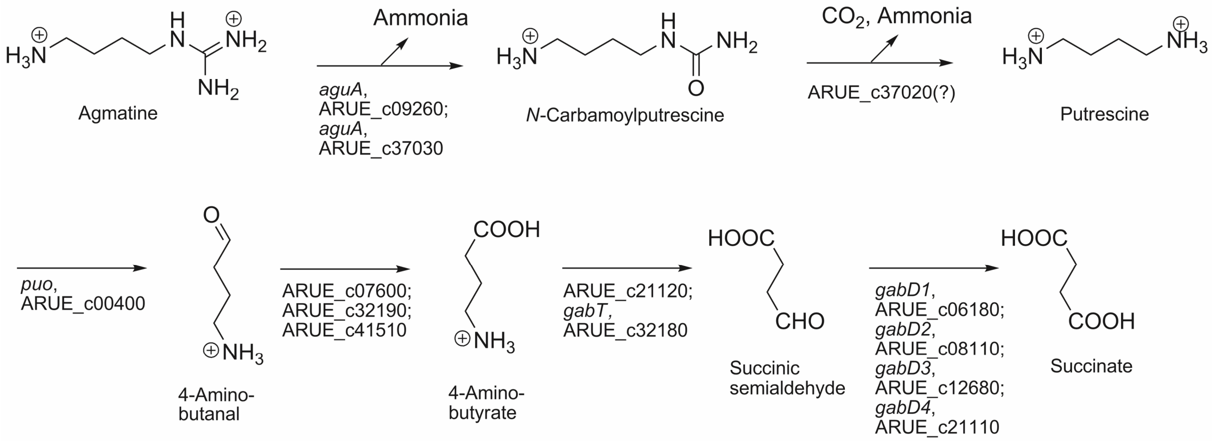

Oxidation of hypoxanthine and xanthine to urate and degradation to allantoin:

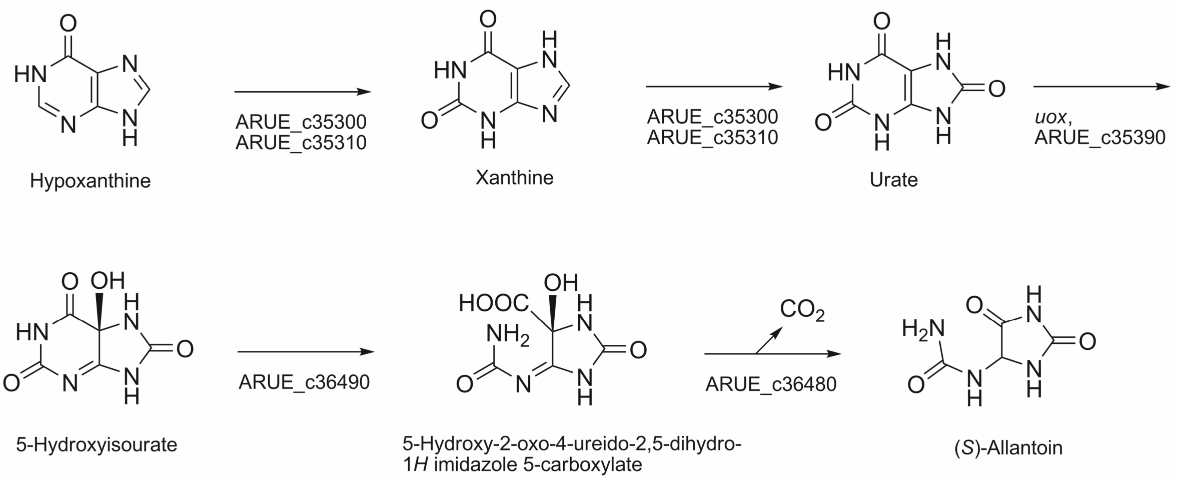

Allantoin degradation to glyoxylate,
glyoxylate metabolism via the D-glycerate pathway:

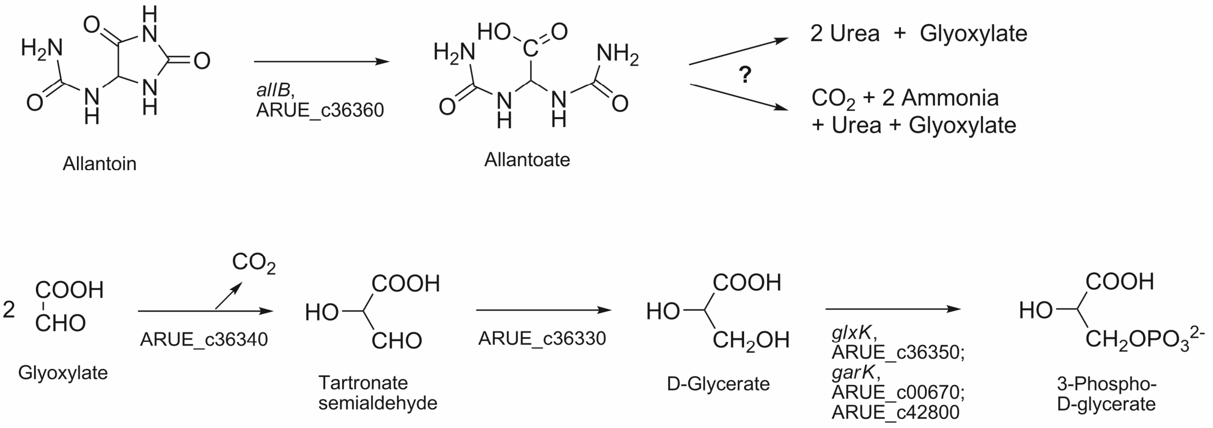

Urea degradation:

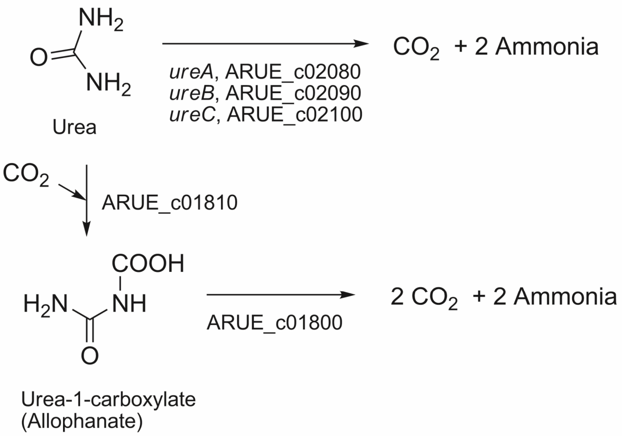

Supplement: Additional file 4 — Figures S1. Biodegradation pathways of Arthrobacter sp. Rue61a. Figures S2. Degradation of 4-hydroxybenzoate, vanillate, and protocatechuate via the ortho pathway. Figures S3. Degradation of 4-hydroxyphenylacetate and homoprotocatechuate via the meta pathway. Figures S4. Choline, creatine and sarcosine metabolism. Figures S5. Agmatine and putrescine degradation. Figures S6. Oxidation of hypoxanthine and xanthine to urate and degradation to allantoin. Figures S7. Allantoin degradation to glyoxylate, glyoxylate metabolism via the D-glycerate pathway. Figures S8. Urea degradation. [file 1471-2164-13-534-S4.pdf]
